# A Combined Experimental/Quantum-Chemical Study of Tetrel, Pnictogen, and Chalcogen Bonds of Linear Triatomic Molecules

**DOI:** 10.3390/molecules26226767

**Published:** 2021-11-09

**Authors:** Freija De Vleeschouwer, Frank De Proft, Özge Ergün, Wouter Herrebout, Paul Geerlings

**Affiliations:** 1 Research Group of General Chemistry (ALGC), Department of Chemistry, Vrije Universiteit Brussel (VUB), Pleinlaan 2, B-1050 Brussels, Belgium; Freija.De.Vleeschouwer@vub.be (F.D.V.); ozgezeynepergun@hotmail.com (Ö.E.); 2 Molecular Spectroscopy Research Group, Department of Chemistry, University of Antwerp (UA), Groenenborgerlaan 171, B-2020 Antwerp, Belgium

**Keywords:** tetrel/pnictogen/chalcogen bonds, quadrupole-dipole model, infrared spectroscopy

## Abstract

Linear triatomic molecules (CO_2_, N_2_O, and OCS) are scrutinized for their propensity to form perpendicular tetrel (CO_2_ and OCS) or pnictogen (N_2_O) bonds with Lewis bases (dimethyl ether and trimethyl amine) as compared with their tendency to form end-on chalcogen bonds. Comparison of the IR spectra of the complexes with the corresponding monomers in cryogenic solutions in liquid argon enables to determine the stoichiometry and the nature of the complexes. In the present cases, perpendicular tetrel and pnictogen 1:1 complexes are identified mainly on the basis of the lifting of the degenerate ν 2 bending mode with the appearance of both a blue and a red shift. Van ′t Hoff plots of equilibrium constants as a function of temperature lead to complexation enthalpies that, when converted to complexation energies, form the first series of experimental complexation energies on sp^1^ tetrel bonds in the literature, directly comparable to quantum-chemically obtained values. Their order of magnitude corresponds with what can be expected on the basis of experimental work on halogen and chalcogen bonds and previous computational work on tetrel bonds. Both the order of magnitude and sequence are in fair agreement with both CCSD(T) and DFA calculations, certainly when taking into account the small differences in complexation energies of the different complexes (often not more than a few kJ mol^−1^) and the experimental error. It should, however, be noted that the OCS chalcogen complexes are not identified experimentally, most probably owing to entropic effects. For a given Lewis base, the stability sequence of the complexes is first successfully interpreted via a classical electrostatic quadrupole–dipole moment model, highlighting the importance of the magnitude and sign of the quadrupole moment of the Lewis acid. This approach is validated by a subsequent analysis of the molecular electrostatic potential, scrutinizing the σ and π holes, as well as the evolution in preference for chalcogen versus tetrel bonds when passing to “higher” chalcogens in agreement with the evolution of the quadrupole moment. The energy decomposition analysis gives further support to the importance/dominance of electrostatic effects, as it turns out to be the largest attractive term in all cases considered, followed by the orbital interaction and the dispersion term. The natural orbitals for chemical valence highlight the sequence of charge transfer in the orbital interaction term, which is dominated by an electron-donating effect of the N or O lone-pair(s) of the base to the central atom of the triatomics, with its value being lower than in the case of comparable halogen bonding situations. The effect is appreciably larger for TMA, in line with its much higher basicity than DME, explaining the comparable complexation energies for DME and TMA despite the much larger dipole moment for DME.

## 1. Introduction

Non-covalent interactions (NCIs) have been playing an increasingly important role in chemistry in recent decades, for example, in the interpretation of the structure of biomolecules and the design of macro- and supramolecular entities. It is sometimes said that, while the 20th century was the century of the covalent bond, the 21st century has become the century of the non-covalent bond. As Schneider formulated it, “with courageous simplification one might assert that the chemistry of the last century was largely the chemistry of the covalent bonding, whereas that of the present century is more likely to be the chemistry of the non-covalent bonding” [1]. Until some decades ago, the variety of non-covalent interactions (NCIs) invoked in most chemical discussions was limited to permanent dipole–permanent dipole, permanent dipole–induced dipole, and induced dipole–induced dipole interactions, also known as the dispersion interaction [2], and, with a unique status, the hydrogen bond. Linus Pauling, to whom this issue of *Molecules* is a tribute, already devoted a separate chapter of his *magnum opus* “The Nature of the Chemical Bond” [3] to the hydrogen bond with concomitant influence in nearly all subfields of chemistry, and over the years, classical textbooks by Pimentel in the 1960s [4] and later on by Schuster [5], Gilli [6], and others have guided generations of theoreticians and experimentalists through its intricacies. Recent reviews (see, for example, [7,8]) pinpointed that its story is still not at the end.

As compared with the other non-covalent interactions, the hydrogen bond has a unique signature comprising the interaction of an electron-deficient H-atom of one molecule (R–H) acting as a bridge to an electron-rich site of another molecule, leading to an attractive Lewis acid–Lewis base interaction. In later years, the spectrum of non-covalent interactions was broadened, essentially by specifying the nature of the dispersion-type interactions, introducing the concept of π–π, σ–σ, and σ–π interactions [9]. The signature of the H-bond stood out until in the early 1990s, an (apparently) new type of NCI where the bridging H-atom is replaced by a halogen atom was scrutinized and baptized the halogen bond [10,11,12,13,14], although halogen bond adducts were known long before (for an historical perspective, see [13]). Although a reversal in the polarity of the R–H bond with partial positive charge on the hydrogen atom, considered as a *conditio sine qua non* for H-bond formation, occurs when introducing a halogen instead of H-atom, a detailed analysis of the charge distribution around the X atom in R–X revealed a highly asymmetric character with, besides a concentration of negative charge in a belt around the bond axis, a buildup of a positively charged region at the opposite side of the R atom. This sigma hole, leading to a positive molecular electrostatic potential [15], was described in detail by Politzer and coworkers [16,17,18,19], and was the basis for rationalizing the strength and directional characteristics of halogen bonds, e.g., increasing strength when passing from F to Cl and via Br to I [10,11,12,13,14]. This picture also fits the existence of the long known (vide supra) halogen bond-adducts “*avant la lettre*” such as I_3_^–^ [20]. An avalanche of papers on both theoretical aspects and applications of halogens bonds have appeared since then, as witnessed in extensive reviews [10,11,12,13,14]. Quantum-chemical calculations, e.g., on structure and stability, are omnipresent, whereas experimental work is less abundant; it concentrates foremost on geometrical aspects of the halogen bond through microwave specroscopy [21,22] and only in a small number of cases an experimental determination of the complexation energy, via an infrared intensity-based method developed in the group of one of the present authors, is reported (vide infra) [23,24,25,26,27,28,29,30,31,32,33,34]. The same characteristics show up in the literature of the next members in this particular series of NCIs, the chalcogen bonding, involving a chalcogen atom (O, S, Se, Te, and so on) (for reviews, see [35,36,37,38]), and the pnic(t)ogen bonding, involving N, P, As, and so on as a substitute for the H atom in a hydrogen bond (for reviews, see [37,38,39,40]). The experimental determination of dissociation constants is, however, limited to the study of the S···O chalcogen bond between C_2_F_4_S_2_ and dimethyl ether (DME) [41].

The next in line [42], the so-called tetrel bond, involving C, Si, Ge, and so on as an H-substitute, has made its appearance as such (i.e., with that name) in a paper by Bauzá et al. [43], finding evidence for close contacts between Si, Ge, and Sn with O or halogen-containing bases and generalizing work by Mani and Arunan [44] on the creation of an electron-deficient site when methane is substituted by an electron-withdrawing group, at the opposite side of the tetrahedron. Tetrel bonds, however, were known long before, but not called as such, for example, in the work by Klemperer et al. [45] in 1984 concluding from the rotational spectrum of CO_2_···NH_3_ a T-shape geometry, and by Newby et al. [46,47] in 2004 coming to similar conclusions on the basis of the rotational spectra of the OCS···DME and CO_2_···DME complexes, in which the latter complex was characterized as dominated by a quadrupole–dipole interaction [48,49]. However, the position of the tetrel bond in the broader context of halogen, chalcogen, and so on bonds was not established yet; as described in several reviews [40,50,51,52], this occurred less than ten years ago, and within that period, a rather intense activity on the description of tetrel bonding flourished. Again, mainly quantum-chemical studies appeared characterizing the geometry and stability of tetrel complexes with an analysis of the bonding itself with a multitude of powerful, well established tools such as EDA [53,54], ELF [55], and so on, accompanied by a limited number of experimental microwave spectroscopy papers [56,57,58] on the determination of the geometrical parameters. Just as in the case of pnictogen bonds, experimental determinations of the stability of these complexes are absent, except, to the best of our knowledge, for the study by the Antwerp group in 2003 on the CO_2_···DME complex [59], yielding a dissociation energy of 16.5 kJ mol^−1^ with a follow-up study on the ν_1_^CO2^/2ν_2_^CO2^ resonance [60] (see Section 2.1.1). An additional exception is work by the same group on the tetrel bond between the complexes of COF_2_ and COFCl with DME described as lone pair···π interactions, but that can be seen as tetrel bonds. However, these complexes refer to sp^2^ carbons with highly polar Lewis acids, which, in view of the sequences to be studied below concerning sp^1^ tetrel (and pnictogen) bonds and the associated electrostatic model involving low or small dipole moments for the Lewis acids, will be left out of consideration in the remaining part of the paper [31].

This situation brought us to the idea of extending the number of tetrel complexes with reliable experimental stability values (see also Section 2.1.1) using a series also allowing direct comparison with pnictogen and chalcogen bonds. The series of three linear triatomics (CO_2_, OCS, and N_2_O) interacting with two donor molecules (trimethyl amine (TMA) and dimethyl ether (DME)) lends itself in an excellent way to such an exercise. As will be seen, they show a variation in (possible) types of bonding (tetrel, pnictogen, and chalcogen), a variation in the geometry of the complex (end-on versus perpendicular), and in one case (OCS) they allow for a direct comparison between chalcogen and tetrel bonding between the two partners. Furthermore, the presence of two donors (TMA and DME) can be used to investigate the influence of the donor. All in all, the choice of these eight complexes permits a variety of comparisons between different types of non-covalent interactions of the “not only” hydrogen bonds (for previous work along these lines, see [51]) and the concomitant interpretations. As will be seen, experimental stability values could be measured for complexes with the three triatomics, be it that the chalcogen bonds with OCS could not be detected. Nevertheless, the six experimental data on tetrel and pnictogen bonds from which five have never been reported, together with the corresponding quantum-mechanical values, form an excellent playground for discussing the (relative) stability of tetrel bonds. The interpretation follows a twofold pattern. First, the data are analyzed in a classical electrostatic model based on the dipole–quadrupole character of the complexes, the way they were characterized in earlier times (vide supra) [45,46,47], accompanied by the analysis of the characteristics of the molecular electrostatic potential [15]. The justification for (the success of) this electrostatic approach is then provided by a quantum-mechanical energy decomposition analysis [53,54], and a further analysis of the electron distribution (and possible charge transfer) is performed via the NOCV (natural orbitals for chemical valence) approach [61], which, particularly in its ETS-NOCV variant [62] (combining NOCV with the extended transition state (ETS) approach [53]), proved to be a highly valuable instrument for characterizing the orbital interactions and concomitant charge transfer as part of the stabilization energy, as, for example, extensively shown by Michalak and coworkers in the study of the σ-hole bonding in halogen bonds [63] and the present authors in the case of the chalcogen bond [36].

The structure of the paper is as follows. In Section 2, the methodology of both the experimental IR measurements and the quantum-chemical calculations will be addressed together with experimental and computational details. In the Results, Section 3, first (in Section 3.1) experimental IR data and the resulting stabilization energies will be reported, and then they are compared with the theoretical stabilization energies (Section 3.2). In the Discussion, Section 4, the electrostatic approach (Section 4.1 and Section 4.3) will be followed by a quantum-chemical justification (Section 4.3) and further analysis of the bonding pattern (Section 4.4). The overall conclusions are presented in Section 5.

## 2. Methodology and Experimental and Computational Details

### 2.1. Infrared Spectroscopy

#### 2.1.1. Methodology

Because of changes in the electron distribution appearing upon complexation, and resulting changes in vibrational frequencies, vibrational spectroscopy in combination with matrix-isolation is a much-used technique for the study of weakly bound molecular complexes. While it cannot compete with the precision and structural definition of gas-phase studies, matrix isolation combined with IR and/or Raman spectroscopy thus provides valuable and often unique information complementary to that derived from gas-phase experiments. Unfortunately, because solid matrices are not in thermodynamic equilibrium, no direct information about the relative stability of the species can be deduced. This shortcoming can be avoided by studying the complexes in cryogenic solutions [64,65,66]. Apart from the relatively low temperatures used during these experiments and the weak solute–solvent interactions present, a major advantage of these cryosolutions is their transparency in a broad spectral interval, ranging from the far-IR to the ultraviolet. As the solutions in general are in thermodynamic equilibrium and often provide the possibility of studying the complexes over a broad temperature range and under equilibrium conditions, they can, in principle, also be used to obtain information about their stoichiometry, their relative stability and, to some extent, even their angular geometry.

During the past two decades, experimental studies in mixed solutions of appropriate Lewis bases and acids in liquid rare gases were investigated using infrared and/or Raman spectroscopy. These have led to a wealth of information on weakly bound molecular complexes involving, among others, the Lewis acids HX (X = F, Cl, Br, and I), BF_3_, and BCl_3_. Solutions in liquefied inert gases have also proven to be an ideal medium to study the spectroscopic and thermodynamic properties of molecular complexes held together by weak red- or blue-shifting C-H···X hydrogen bonds or weak and medium-strong C−X···Y (with X = I, Br, Cl and Y = O, N, S, F, Cl, *π*, and so on) halogen bonds. The experimental setups for the infrared and Raman studies of cryosolutions and general methodologies used to characterize these molecular complexes observed have been reviewed in depth [64,65,66,67], and will thus not be discussed in detail here.

Triggered by the original work of Klemperer and co-workers, [45] cryosolutions have also been used to characterize the 1:1 complex formed between dimethyl ether and CO_2_. In the original study by Van Ginderen et al., the 1:1 complex was studied experimentally in solutions in liquid argon (LAr), using infrared spectroscopy, and theoretically, using ab initio calculations at the MP2/6-311++G(d,p) level [68]. The complex was found to be formed through the interaction of the dimethyl ether oxygen atom with the CO_2_ carbon atom, with the observed vibrational bands being in good agreement with the theoretically predicted vibrational frequencies. In addition, the standard complexation enthalpy of the complex in LAr was determined from a temperature-dependent study of the spectra and was found to be −8.0(3) kJ mol^−1^.

It should be stressed that, although the complexes of dimethyl ether with ^12^CO_2_ and ^13^CO_2_ reported in the previously mentioned studies can now be identified as tetrel bonded complexes, this idea was not pursued in the earlier studies. Realizing the renewed interest in tetrel, pnictogen, and chalcogen bonded interactions, in this study, we expand the available experimental data on tetrel bonded complexes by adding cryospectroscopic data for the complexes of CO_2_ with trimethyl amine, which is generally accepted to be a stronger Lewis base than dimethyl ether. In addition, to shed further light on the nature of pnictogen and chalcogen bonding, and the competition of the latter with tetrel bonding, for both dimethyl ether and trimethyl amine, additional studies on the complexes formed with nitrous oxide, N_2_O, carbon disulfide, CS_2_, and carbonyl sulfide, OCS, were initiated, as stated in the Introduction. For both N_2_O and OCS, complexes could indeed be identified and fully characterized. Unfortunately, owing to the limited solubility of CS_2_ in liquid rare gases, no experimental data on its complexes with dimethyl ether or trimethyl amine could be derived.

Before discussing the actual results in Section 3.1 and Section 4, it is worth noting that the cryosolutions typically create a weakly interacting environment that, when combined with the low temperatures used, leads to small band widths. This is of utmost importance in this study, where owing to the weakness of some of the complexes studied, new bands are often only slightly shifted from the corresponding monomer bands and complex and monomer bands often show considerable overlap. To separate the contributions from the monomers and complex, subtraction techniques are thus required, in which spectra of the monomer solutions, recorded at similar concentrations and at identical temperatures, are rescaled and subtracted from the spectra of the mixture. Using subtracting procedures, the band areas of the monomer and complex bands can be accurately determined using a simple numerical integration, so that the inaccuracies inherently connected to resolving strongly overlapping bands with least square band fitting procedures can be avoided. This result is important, as accurate band areas of monomer and of complex bands are required in confirming the stoichiometry of the complex and in determining the standard complexation enthalpy. In some cases, the use of subtraction procedures also allows weak spectral features not immediately visible in the original spectra to be identified.

The standard complexation enthalpies in liquid argon are traditionally derived from temperature studies in which spectra of a solution are recorded at a variety of temperatures, and in which the resulting band areas of monomer and complex bands of different solutions are analyzed using the Van ′t Hoff relation. The key element of this analysis is the approximation that, in limited temperature intervals, the standard complexation enthalpies and the corresponding values for the complexation entropies are independent of temperature. For traditional solvents, the complexation enthalpy Δ*H*^0^ for a complex *A_m_B_n_* can be obtained by plotting the logarithm of the intensity ratio of complex and monomer intensities versus the inverted temperature, 1/T, with the relation between both quantities, derived from the Van ′t Hoff isochore, being given as follows:

(1) $$\ln\left[\frac{I_{A_{m}B_{n}}}{I_{A}^{m}\times I_{B}^{n}}\right]=-\frac{\Delta H^{0}}{RT}+c^{st}$$

This approach is valid when the temperature variation of the spectral intensities is fully accounted for by the shift in the chemical equilibrium as a consequence of a change in temperature. For cryo-solutions such as solutions in liquid rare gases, this condition is not fulfilled, because of the pronounced variation in liquid density of these solvents over the available temperature interval. Therefore, additional corrections are required [67]. Although solutions in liquefied rare gases were originally described as a pseudo-gas phase, it is now generally accepted that, in the cryo-solutions, significant solute–solvent interactions can occur. These interactions not only influence the frequencies of monomers and complexes, but also perturb the relative stability of the complexes studied. The experimental values derived from cryo-solutions should thus not be directly compared with ab initio complexation energies or vapor phase complexation enthalpies. To correct for solvent effects, and to obtain a vapor phase complexation enthalpy, for all species involved, the solvation processes in liquid rare gases were simulated using Monte Carlo-based free energy perturbation calculations using the same procedures as those described before [64,65,66].

To support the experimental measurements, harmonic vibrational frequencies and infrared intensities of monomers and complexes were obtained from MP2/6-311++G(d,p) ab initio calculations [68], using Gaussian 09 [69]. During all calculations, corrections for the basis set superposition error (BSSE) [70] were accounted for explicitly using Counterpoise-corrected gradient techniques [71,72]. The resulting values are summarized in Tables S10–S17 of the ESI.

#### 2.1.2. Experimental Details

All samples were purchased from Sigma-Aldrich (Sigma-Aldrich, Bornem, Belgium). The samples were transferred into glass sample tubes and degassed using a freeze–thaw cycle procedure. The solvent gas argon was supplied by Air Liquide and had a stated purity of 99.9999% (Air Liquide, Herenthout, Belgium).

The IR spectra were recorded on a Bruker 66v FTIR spectrometer, equipped with a Globar source, a Ge/KBr beam splitter, and MCT detector (Bruker Optics, Ettlingen, Germany). Measurements were conducted in cells equipped with Si windows and a path length of 10 mm to obtain spectra between 4500 cm^−1^ and 450 cm^−1^. All interferograms were averaged over 500 scans, Blackman–Harris three-term apodized, and Fourier transformed to yield spectra with a resolution of 0.5 cm^−1^. Estimated mole fractions of the solutions varied between 1 × 10^−5^ and 1 × 10^−3^ for the Lewis bases DME and TMA and between 1 × 10^−6^ and 8 × 10^−4^ for the Lewis acids ^12^CO_2_, ^13^CO_2_, N_2_O, and OCS. As the experimental setups do not allow for verification of full solubility of the compounds, or verification of the fluid level in the filling tube [64,65,66], exact concentrations are not known.

### 2.2. Theory and Computational Details

Ab initio calculations using Gaussian 16 [73] were carried out to determine, on the one hand, the stability of the aforementioned complexes and, on the other hand, properties relevant to this study such as the dipole and quadrupole moments and the molecular electrostatic potential. Equilibrium geometries and harmonic vibrational frequencies for monomers and complexes were obtained using the full MP2/aug-cc-pVTZ level of theory [74,75]. The vibrational analyses showed that the optimized structures are exclusively characterized by positive eigenvalues, and thus represent minima on the potential energy surface. Refined electronic energies were determined for the CCSD(T) method by extrapolating the coupled-cluster energies to an asymptotically complete basis set (CBS). More specifically, the three-point extension of the Schwartz extrapolation formula [76,77]:

(2) $$E_{corr}\left(l\right)=E_{corr}\left(\infty \right)+\frac{B}{{\left(l+1/2\right)}^{4}}+\frac{C}{{\left(l+1/2\right)}^{6}}$$

was employed to retrieve the correlation energy at the CBS limit, together with the Feller three-point extrapolation of the Hartree–Fock energies [78,79]:

(3) $$E_{HF}\left(l\right)=E_{HF}\left(\infty \right)+Ae^{-Bl}$$

with $E_{HF}\left(\infty \right)$ also written as $F\left(\infty \right)$ and the cardinal *l* number equal to 2, 3, 4, 5, and so on when X = D, T, Q, 5, and so on using the cc-pVXZ basis set. In this work, the employed Feller and Schwartz extrapolation procedures are made of data obtained using the cc-pVDZ, cc-pVTZ, and cc-pVQZ basis sets [74]. The CCSD(T) energy in the limit of an asymptotically CBS, *S*_CCSD(T)_, can then be calculated as follows:

(4) $$S_{CCSD(T)}\left(\infty \right)=E_{HF}\left(\infty \right)+E_{corr}\left(\infty \right)$$

Besides the wavefunction methods, a small-scale benchmark on the experimental complexation energies using a variety of DFT functionals was performed. Basis set superposition error (BSSE) [70] was accounted for using the Counterpoise correction [71,72]. The full benchmark results are collected in ESI, Table S3, and demonstrate the excellent performance of M06-2X/cc-pVTZ [74,75,80], with Grimme’s D3 dispersion correction [81] added, for our set of non-covalent interaction energies.

Finally, Ziegler–Rauk-type energy decomposition analyses (EDA) [53,54], together with the natural orbital for chemical valence (NOCV) approach [61,62,63], were applied to all investigated complexes to shed light on which stabilizing energy terms dominate the interaction energies and whether charge transfer is a contributing factor. The PBE0 hybrid functional [82] in combination with the TZ2P (small core) basis set and Grimme′s D3 method with Becke–Johnson damping [83], as implemented in ADF2020 [84,85], was selected as the level of theory. Relativistic effects were included via the zeroth-order regular approximation (ZORA) [86,87]. The complexation energy is first subdivided into a strain term, i.e., the deformation energy needed for the isolated monomers to adopt their geometry in the complex, and the interaction energy between the fragments in the complex. The interaction energy can then be further decomposed into a destabilizing term; the Pauli repulsion caused by the repulsive interactions between occupied orbitals; and three stabilizing terms, the classical electrostatic interactions between the charge densities of the two monomers, the orbital interactions accounting for electron pair bonding, charge transfer and polarization, and a dispersion term representing the van der Waals interactions between the two fragments:

(5) $$\Delta E_{int}=\Delta E_{Pauli}+\Delta V_{elst}+\Delta E_{oi}+E_{disp}$$

Additional insight into the specific orbital interactions in the complex can be obtained through NOCVs, defined as the eigenvectors that diagonalize the deformation density Δρ(**r**). The ETS-NOCV approach allows for a breakdown and visualization of the reorganization of the charge distribution between the monomers upon complexation.

## 3. Results

### 3.1. Infrared Spectroscopy

**Figure 1:**
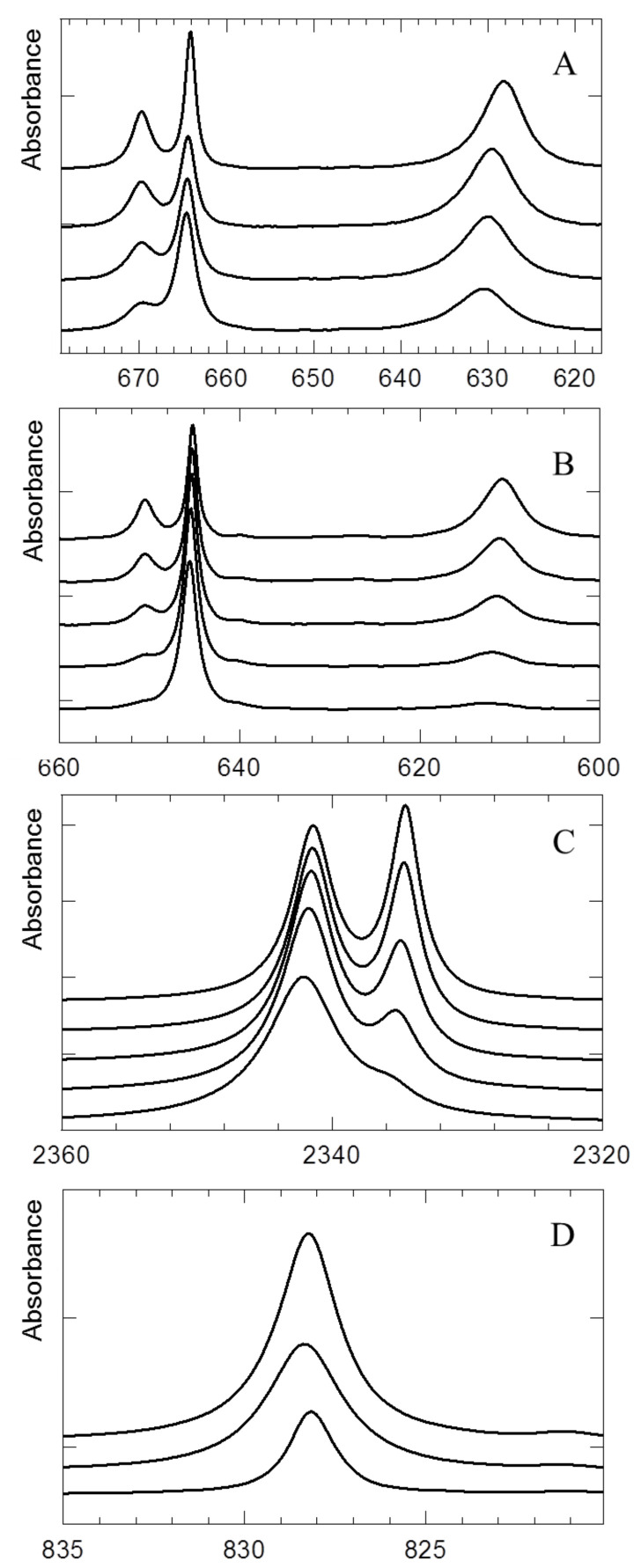
Spectral data obtained for solutions in liquid argon-containing mixtures of trimethyl amine with ^12^CO_2_ or ^13^CO_2._ More details can be found in the text.

Typical results obtained for solutions in liquid argon containing mixtures of trimethyl amine and ^12^CO_2_ or ^13^CO_2_ are collected in Figure 1. In panels A and B, the spectral features illustrating the effect of complexation on the originally degenerated ν_2_ bending vibrations in ^12^CO_2_ or ^13^CO_2_, respectively, are summarized, showing the lifting of the degeneracy with concomitant blue and red shifts. Spectral features illustrating the effect of complexation on the ν_3_ antisymmetric stretching vibration in ^12^CO_2_ are given in panel C. The estimated mole fractions used for panels A to C are 4 × 10^−4^ for TMA and 4 × 10^−6^ for ^12^CO_2_ and ^13^CO_2_. For those panels, spectra were recorded at different temperatures between 93 and 113 K, with the spectra obtained at the lowest and highest temperatures being shown in the upper and lower trace, respectively. Panel D shows the results of a spectral decomposition analysis for the ν_6_ region of TMA obtained for a mixed solution containing approximate mole fractions of 3 × 10^−5^ for TMA and 6 × 10^−6^ for CO_2_. The original spectrum recorded at 97 K is shown in the upper trace. The middle and bottom trace refer to the rescaled spectrum of monomer TMA recorded at the same temperature, and to the difference spectrum showing the contributions of the complex with CO_2_, respectively. As before, solutions are recorded at different temperatures between 93 and 113 K, and spectra are shown with increasing temperature.

**Figure 2:**
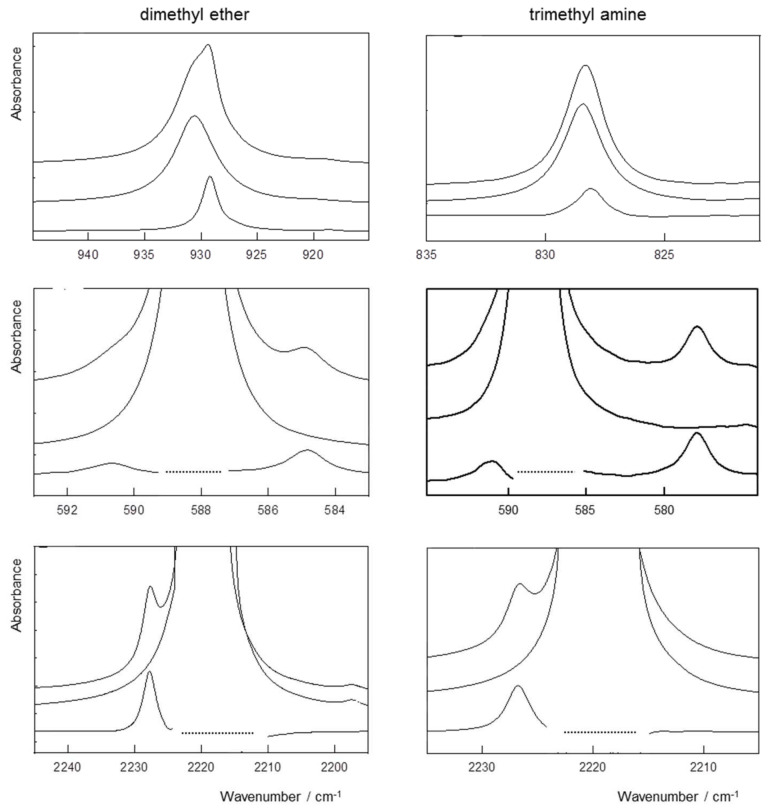
Spectral decomposition analyses obtained for the spectra of solutions in LAr-containing mixture of N_2_O and dimethyl ether (**left**) and of N_2_O and trimethyl amine (**right**). More details can be found in the text.

Characteristic features obtained for solutions in liquid argon-containing dimethyl or trimethyl amine and N_2_O are reported in Figure 2. The upper panels refer to the ν_6_ spectral region of DME and TMA, respectively. The middle and bottom panels show the results for the ν_2_ bending vibration and the ν_3_ antisymmetric stretching mode, respectively. For each panel, the original spectrum recorded at 97 K is shown in the upper trace. The middle and bottom trace refer to the rescaled spectrum of monomer DME or TMA recorded at the same temperature and to the difference spectrum showing the contributions of the complex with N_2_O, respectively. For both Lewis bases, a blue shift is observed for the ν_3_ antisymmetric stretching mode. In addition, the degeneracy of the ν_2_ bending vibration in N_2_O is lifted, with two new bands due to the modes in the complexes appearing blue and red shifted from the monomer frequency. The estimated mole fractions used for the spectra in the upper panels are 6 × 10^−4^ for TMA or DME and 5 × 10^−4^ for N_2_O, whereas those used for the spectra in the bottom panels are 4 × 10^−4^ for TMA, 5 × 10^−3^ for DME and 3 × 10^−5^ for N_2_O. The dotted lines shown in the middle and bottom panels are used to mark the spectral regions for which the original spectra are perturbed by the non-linearity of the MCT detector and for which no reliable data for the complex can thus be derived.

**Figure 3:**
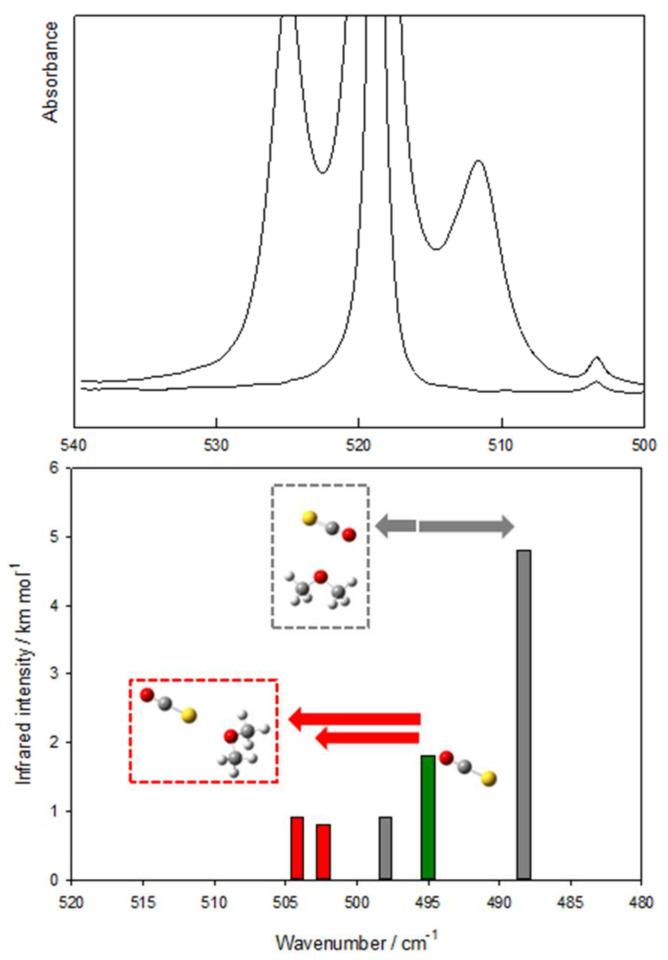
Typical spectra obtained for solutions in LAr at 123 K containing mixtures of OCS and dimethyl ether. More details can be found in the text.

Spectral data for the complexes of dimethyl ether and OCS, allowing the nature of the complex to be identified as tetrel bonded, are summarized in Figure 3. The top panel displays the experimental spectra showing the ν_2_ OCS bending vibration for a mixed solution containing dimethyl ether and OCS and that for a solution containing only OCS. The estimated mole fractions are 1 × 10^−3^ for DME and 1 × 10^−4^ for OCS. To allow the complex to be identified as tetrel bonded, in the lower panel, the calculated frequencies for the OCS monomer and for the tetrel and chalcogen bonded isomers of the 1:1 complex are compared. In agreement with the results for CO_2_ and N_2_O, for OCS, the degeneracy of the ν_2_ bending vibration is also lifted, with one component being blue shifted from the monomer and the other one being red shifted. These trends are in line with the computed predictions for the tetrel bonded complex, but contrast with those obtained for the chalcogen bonded isomer. This proves that, in solutions, only the tetrel bonded isomer of the complex is formed.

**Figure 4:**
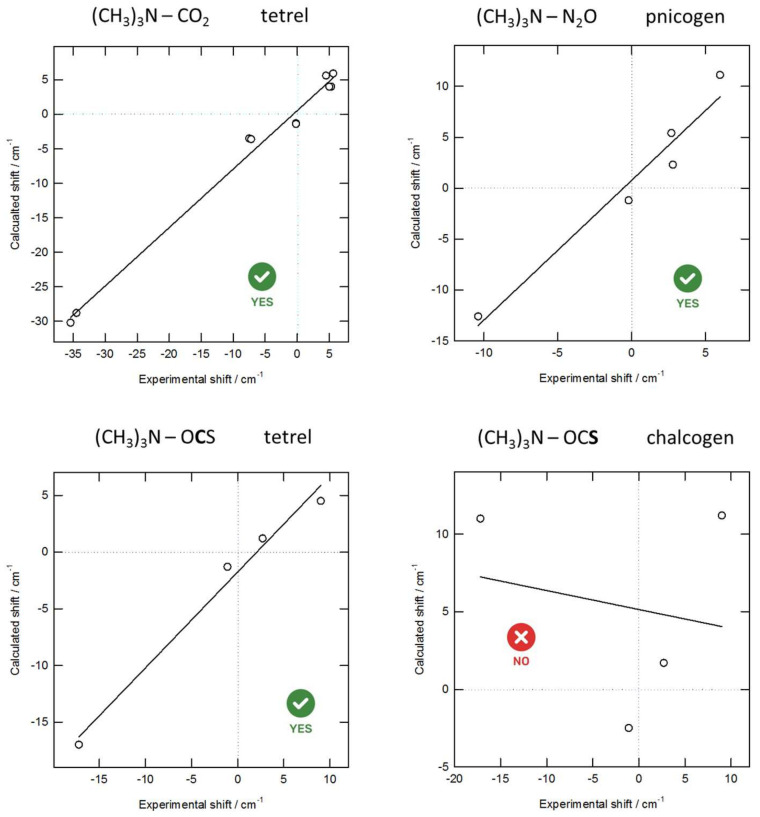
Scatter plot showing the most important (ν_2_ OCS, ν_6_ TMA, and so on) experimentally observed complexation shifts with TMA and the calculated values derived from the ab initio harmonic vibrational frequencies. The solid line represents the linear regression line.

**Figure 5:**
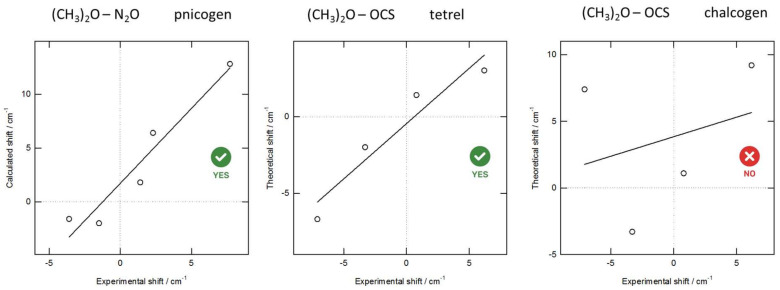
Scatter plot showing the experimentally observed complexation shifts with DME and the calculated values derived from the ab initio harmonic vibrational frequencies. The solid line represents the linear regression line.

The observed experimental frequencies for the monomers and for the complexes, their assignment, and the complexation shifts deduced are summarized in Tables S4–S9 in the ESI. Calculated harmonic frequencies and intensities with the frequency shifts are given in Tables S10–S17 of the ESI. The scatter plots of experimentally observed and calculated complexation shifts in Figure 4 and Figure 5 allow for the identification of the nature of the observed complexes.

For correct assignments, the linear regression line, correlating the experimental observations with the computed complexation shifts, should have a slope close to one and should cross, within error margins, the origin of the chosen coordinate system. A good correlation is found for the N···C tetrel and N···N pnictogen bonded complexes between TMA and CO_2_ or OCS and TMA and N_2_O, respectively. In contrast, for the complex of TMA with OCS, little or no correlation is observed when the calculated values for the N···S chalcogen bonded isomer are used. These findings are confirmed by the scatter plots for DME as Lewis base in the complexes with N_2_O and SCO (Figure 5).

To summarize, mainly the observed lifting of the degeneracy of the CO_2_, N_2_O, and OCS ν_2_ bending mode, with concomitant blue and red shift, as well as the shift of the ν_3_ antisymmetric stretching of CO_2_ and N_2_O and the ν_6_ (CO/CN) stretching frequency shifts of DME and TMA in bonds close to the lone pair(s) carrying O and N atoms of the bases, lead to the unambiguous assignment of the bonding pattern in all complexes, both with DME and TMA, as perpendicular (tetrel or pnictogen), and strongly indicate the absence of a chalcogen complexation in, e.g., OCS.

**Figure 6:**
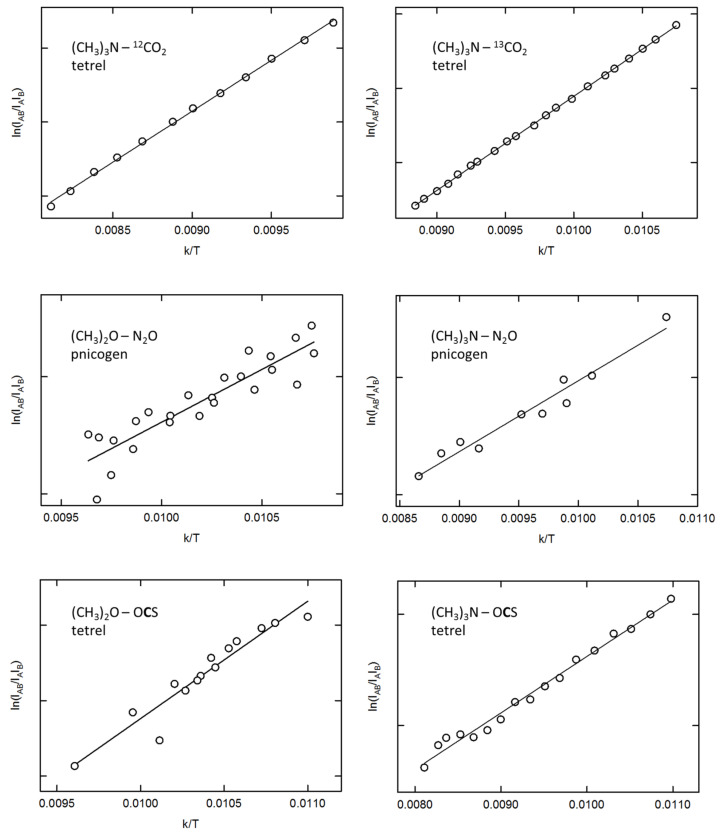
Van ′t Hoff plots for determining the complexation enthalpies for the various complexes observed.

Lastly, experimentally derived vapor phase complexation energies are determined for six of the complexes, being the tetrel bonds between CO_2_/OCS and DME/TMA and the pnictogen bonds between N_2_O and both Lewis bases. The Van ′t Hoff plots for the various complexes are given in Figure 6. The resulting complexation enthalpies for liquid argon, $\Delta H_{LAr}^{0}$, in Table 1 are obtained by calculating the corresponding regression lines and correcting the slopes for changes in liquid density. The vapor phase complexation enthalpies, $\Delta H_{vapor}^{0}$, are derived by correcting the solution phase data for solvent effects, using data from free-energy perturbation-based Monte Carlo simulations [88]. Finally, corrections for the vapor phase complexation enthalpies using rigid rotor/harmonic oscillator based statistical thermodynamical models yield the vapor phase complexation energies, $\Delta E_{exp}$.

The order of magnitude of the experimental values is in line with what could be expected on the basis of previous experimental work for halogen bonds for which a variety of data exists [23,24,25,26,27,28,29,30,31,32,33,34] (vide supra), and for chalcogen bonds where only a single experiment was carried out [41]. Moreover, these values are also consistent with computational data for which much more material is available in the literature and whose trends (see, for example, Scheiner′s review [51]) at first glance largely parallel the observed tendencies for cases showing a variety in terms of type of bond (tetrel, pnictogen, and chalcogen) and geometry (end-on and perpendicular). Its detailed discussion will be given in Section 4. Note that all this should be viewed in the context of the low stability values (at most, 20 kJ mol^−1^), with experimental errors based on the standard deviation on the slope of the regression lines varying between 0.4 and 1.5 kJ mol^−1^.

### 3.2. Ab Initio and DFA-Based Complexation Energies

**Figure 7:**
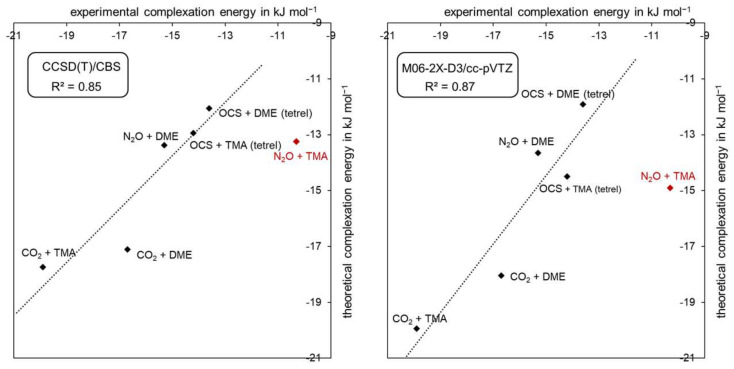
Correlation between theoretical and experimental complexation energies. Left: CCSD(T)/CBS. Right: M06-2X-D3/cc-pVTZ. The dashed line represents the linear regression line. Pnictogen complex N_2_O + TMA is excluded from the linear regression analysis. The linear regression for all six complexes results in R^2^ values of 0.61 for CCSD(T) and 0.51 for M06-2X levels of theory.

Table 2 lists the complexation energies at 0 K and excluding the zero-point energy for all eight complexes computed with (1) BSSE-corrected MP2/aug-cc-pVTZ, i.e., the level of theory selected for the geometry optimizations; (2) CCSD(T) at the complete basis set limit (CBS); and (3) the best-performing DFT functional M06-2X-D3/cc-pVTZ, from a benchmark study reported in the ESI (Table S3). Figure 7 depicts the linear correlation between the experimental and theoretical complexation energies, with CCSD(T)/CBS shown on the left and M06-2X-D3/cc-pVTZ on the right. Note that the N_2_O···TMA complex could be considered as an outlier. In view of the low number of data points and the experimental error (Table 1), we report the statistics both with and without inclusion of this point.

In summary, it can be stated that the agreement between the theoretical and experimental values is good to excellent, both at the CCSD(T)/CBS wave function “golden standard” and with DFT using the M06-2X-D3 functional, with mean unsigned errors of 2.1 and 1.6 kJ mol^−1^, or 1.5 and 1.0 kJ mol^−1^, respectively, when discarding the N_2_O···TMA complex and correlation coefficients of 0.85 and 0.87, again excluding the N_2_O···TMA complex. Remarkably, the performance of the Counterpoise-corrected MP2/aug-cc-pVTZ method is similar to the CCSD(T)/CBS level of theory, and the M06-2X-D3 density functional approximation (DFA) performs even better than the CCSD(T) level. However, this is not the case for all DFAs, as witnessed from the small benchmark study in the ESI (Table S3). Using other dispersion-corrected and/or range-separated functionals results in errors that are three- to even sixfold that of M06-2X-D3. Nonetheless, adding dispersion is vital for treating non-covalent interactions, as many literature studies on the topic have already dictated and as confirmed by the poor results of the PBE0/cc-pVTZ method.

Note also in this discussion that one treats energetics below the overall accepted 1 kcal mol^−1^ limit of chemical accuracy and that, taking into account the experimental errors, the overall agreement, i.e., including the N_2_O···TMA complex, can be considered as very good. These data, therefore, can be considered to form a sound basis to have an in-depth discussion on their variation throughout the complete series for which, consistently, the theoretical values will be selected in view of the absence of two experimental data.

## 4. Discussion

### 4.1. The Quadrupole-Dipole Model

As stated above, we initiate the discussion based on electrostatic arguments using the dipole and quadrupole moments of the interacting species. Table 3 shows the comparison between the experimental values and the theoretical dipole and quadrupole values obtained at the MP2/aug-cc-pVTZ level of theory. Note that, in the case of non-zero dipole moment molecules, the quadrupole moment data refer to center-of-mass values.

Table 3 shows that the agreement between theoretical and experimental values is excellent both in sign and magnitude. Therefore, in the following discussion, where mostly trends and signs are at stake, no particular preference for experimental or theoretical values exists as long as they are used consistently.

Returning now to the sequence of experimental stability data, parallel with and completed by the theoretical data in Table 2, the most stable complexes turn out to be the CO_2_ ones (DME···CO_2_ and TMA···CO_2_), for which the smallest Lewis acid–Lewis base distances are registered (see Table 4). More importantly, in each case, a perpendicular geometry was observed (T shape), in line with the early experimental data by Newby on the DME···CO_2_ complex [47] and the above-mentioned 1984 study by Klemperer et al. [45] on a simpler congener of the TMA···CO_2_ complex, the NH_3_···CO_2_ complex, without, however, yet referring to a tetrel complex. These complexes are similar in nature to the observed OCS tetrel complexes and the pnictogen complexes of N_2_O, all having a T shape geometry. The theoretical calculations corroborate these observations, with only minor deviations from the perpendicular geometry with angles ranging between 85° and 92°, as listed in Table 4.

The origin of this shape can be traced back to the quadrupole–dipole nature of these complexes. Indeed, concentrating first on the CO_2_ complexes, in the absence of a dipole moment of this Lewis acid, the dipole–dipole interaction between Lewis acid and Lewis base is absent and, therefore, the interaction between this molecule and the Lewis bases (TMA and DME) can be described by an interaction between the quadrupole of CO_2_ and the dipole of the Lewis base (where, for the sake of simplicity, the N lone pair moment or the resultant of the O lone pairs moment can be considered as being representative for it). Going back to Buckingham’s masterly theoretical treatises in [48,49], more particularly, Equation (23) of [48] or Equation (1.157) in [49], the electrostatic interaction energy between two charge distributions is decomposed, as given below in Equation (6):

(6) $$\begin{array}{cc} \Delta E_{12}\approx \frac{1}{R}q_{1}q_{2} & +\frac{1}{R^{2}}f\left(q_{1}\mu_{2},q_{2}\mu_{1}\right)+\frac{1}{R^{3}}\left[f\left(q_{1}\Theta_{2},q_{2}\Theta_{1}\right)+c\mu_{1}\mu_{2}\right]\\ & +\frac{1}{R^{4}}\left[f\left(q_{1}\Omega_{2},q_{2}\Omega_{1}\right)+f\left(\mu_{1}\Theta_{2},\mu_{2}\Theta_{1}\right)\right]+\frac{1}{R^{5}}c\prime \Theta_{1}\Theta_{2}+\ldots \end{array}$$

with *q* being the charge, μ being the dipole moment, Θ being the quadrupole moment, and Ω being the octopole moment, and where the *f* functions also depend on geometrical parameters. For the Lewis base···CO_2_ complex, with the Lewis base denoted as charge distribution 1 and CO_2_ as charge distribution 2, this equation reduces to the following:

(7) $$\Delta E_{12}\approx \frac{1}{R^{4}}c^{\prime \prime }\mu_{1}\Theta_{2}+\frac{1}{R^{5}}c\prime \Theta_{1}\Theta_{2}+\ldots \;$$

The resultant R^–4^ interaction displays a proportionality to the magnitude of the dipole moment of the Lewis base and the quadrupole moment of the Lewis acid of the interacting systems. This product is multiplied by a geometrical factor ($c^{\prime \prime }$ in Equation (7)), whose analysis leads to the conclusion that the most stable configurations are [48], in the case of a negative Q (denoted as Θ by Buckingham) value, either the perpendicular T shape geometry or an end-on geometry as in the chalcogen bond, in which, however, the dipole orientation should be reverted, evidently yielding in our case a less stable situation.

The sign of Q is thus of fundamental importance and already indicates that, for a given constant μ value (so either considering DME or TMA), the more negative Q value should be accompanied by a higher stability of the complex, as is indeed found when comparing the stability of the tetrel CO_2_ complexes (Q = −4.28) to that of the OCS complexes (Q = −0.78). Interestingly, although the complexes of N_2_O are different in the nature of the central atom of the Lewis acid (N vs. C, so pnictogen versus tetrel bonding), they can in the case of these π-hole bonding interactions (vide infra) be treated in a similar way. On the basis of the Q value for N_2_O (−3.30), an intermediate stability for these complexes is expected, as indeed turns out to be the case. For N_2_O, the perpendicular arrangement of the two complexes DME···N_2_O and TMA···N_2_O is in agreement with that of their simpler congeners NH_3_···N_2_O and H_2_O···N_2_O concluded in early work by Klemperer [95,96]. In the last two cases (N_2_O and OCS), it should be remarked that both molecules do have a non-zero dipole moment, but that the perpendicular orientation in the complexes of the Lewis acid and Lewis base dipole moment reduces the dipole–dipole interaction energy to zero, as can be seen from an analysis of the geometrical factor in the R^−3^ interaction term between two permanent dipole moments at a fixed geometry [48,49] (the well-known R^−6^ dependence showing up only after Boltzmann averaging). Passing to positive Q values, as in CS_2_ and CSe_2_ (Table 3), is expected to yield no stable tetrel complex, as the interaction energy would then turn out to be positive. A recent microwave study on the complex between CS_2_ and NH_3_ by Legon et al. indeed yielded evidence for a chalcogen bond with an end-on geometry without any indication of the existence of a tetrel bond [97]. The quadrupole moments for higher chalcogen analogues of CO_2_, of the type CBB’ (B, B’ = O, S, Se, and Te) as calculated in [98] at CCSD(T) level and in the present work, invariably are positive preventing further possibilities of tetrel bonding in T shape complexes, with the only exception being OCS, its “lowest” analogue.

Focusing on the difference between the interaction energies for DME and TMA in the T shape complexes, it should be remarked that the difference in their dipole moments is not reflected in the interaction energies and that, at first sight, remarkably, the interaction energies with TMA are comparable and sometimes even undeniably higher than with DME, which is opposite to their outspoken dipole moment sequence. The same observation was made for the halogen bonding of both Lewis bases with CH_2_FI and CHF_2_I (see [32]). This observation has to be placed next to the well-known fact [99] that amines are stronger bases than ethers and that, in this context, the charge-transfer contribution to the interaction energy plays an extra role when discussing the difference in interaction energies between TMA and DME (vide infra in the EDA and NOCV analysis).

Coming back to the OCS case, the combined results for a chalcogen and a tetrel bond indicate, depending on the theoretical method used, a stronger interaction for the tetrel bond (with TMA and DME) for the M06-2X-D3 functional (and the benchmark functionals ωB97X-D, B97X-D, and M11), or a similar interaction (in the case of TMA) for the MP2 and CCSD(T) methods (and the benchmark functional PBE0-D3BJ). Experimentally, only the tetrel bonded complexes were detected (vide supra). This finding has to be confronted with the overall conclusion by Scheiner in his analysis of the relative strengths of the H-bond analogues, pointing to a chalcogen bond as being stronger than a pnictogen and tetrel bond, which are of comparable strength [51]. However, this analysis is based on Lewis acids of the type HBr, H_2_S, H_3_As, and H_4_Ge, where substitution of H by F or methyl sometimes influences the sequences, but where the essential point is that these are all σ-complexes. The tetrel and pnictogen complexes considered in the present work are π-complexes, exploiting the π-hole (vide infra). Possible steric hindrance when exploiting the σ-hole is largely avoided in the perpendicular T shape geometry. The experimental observations in this study are also not in agreement with a recent publication by Legon et al. [100], in which the authors reported for the simpler congener of the TMA···OCS complex, i.e., the OCS···NH_3_ complex, a symmetric top structure pointing to a chalcogen bond at the S atom. On the other hand, Alkorta et al. [101] made an extensive computational study on chalcogen and tetrel bonds between OCS and different nitrogen bases (with sp^1^ and sp^2^ hybridized N atoms) in which the preference for chalcogen or tetrel bonds varies from case to case, probably depending on secondary factors. OCS complexes with para-substituted pyridines were extensively studied by Chandra, Zeegers-Huyskens et al., showing very similar interaction ranges for the two types of bonds [102].

All in all, in the case of OCS with its low quadrupole moment, there seems to be competition between the two types of bonding, where, probably, sometimes a delicate equilibrium is established. The experimental data in our study, however, unambiguously point to a tetrel complex, without indication of a chalcogen complex. This absence in the experimental observations (on the basis of enthalpy and energy considerations) of an at first sight perfectly feasible complex was also noticed in previous experimental work by the Antwerp group in the case of the competition between halogen and hydrogen bonding [32,34]. This phenomenon could tentatively be assigned to effects of the entropy of solvation, which are different in the two complexes, certainly in the present case owing to their fundamentally different geometries.

Finally, note that, despite its much smaller quadrupole moment compared with N_2_O, the tetrel bond in OCS is only slightly weaker than the pnictogen bond of N_2_O. It could be that the appreciably larger dipole moment of OCS, as compared with the near-zero value of N_2_O, is involved in a dipole–quadrupole interaction with now the quadrupole of the base, leading to an overall similar electrostatic interaction present in both complexes (see also the EDA analysis in Section 4.3).

### 4.2. Molecular Electrostatic Potential

The foregoing discussion has been focusing on classical electrostatic arguments and it should obviously be coupled to the ansatz that is now mostly found in the discussion of the strength and structure of H-bond congeners, which is purely quantum-chemical and in which the concept of σ-and π-holes [16,17,18,19,37,103] stands central. These concepts were introduced and later on quantified by Politzer and coworkers and, in this endeavor, the detailed consideration of the molecular electrostatic potential (MEP) surface, both qualitatively and quantitatively, played an elusive role [16,17,18,19]. Moreover, the consideration of the importance of the MEP is a first, rough, approach for estimating the role of electrostatic effects upon complexation and the study of the energy decomposition analysis afterwards will be used to judge the adequateness of this line of reasoning.

**Figure 8:**
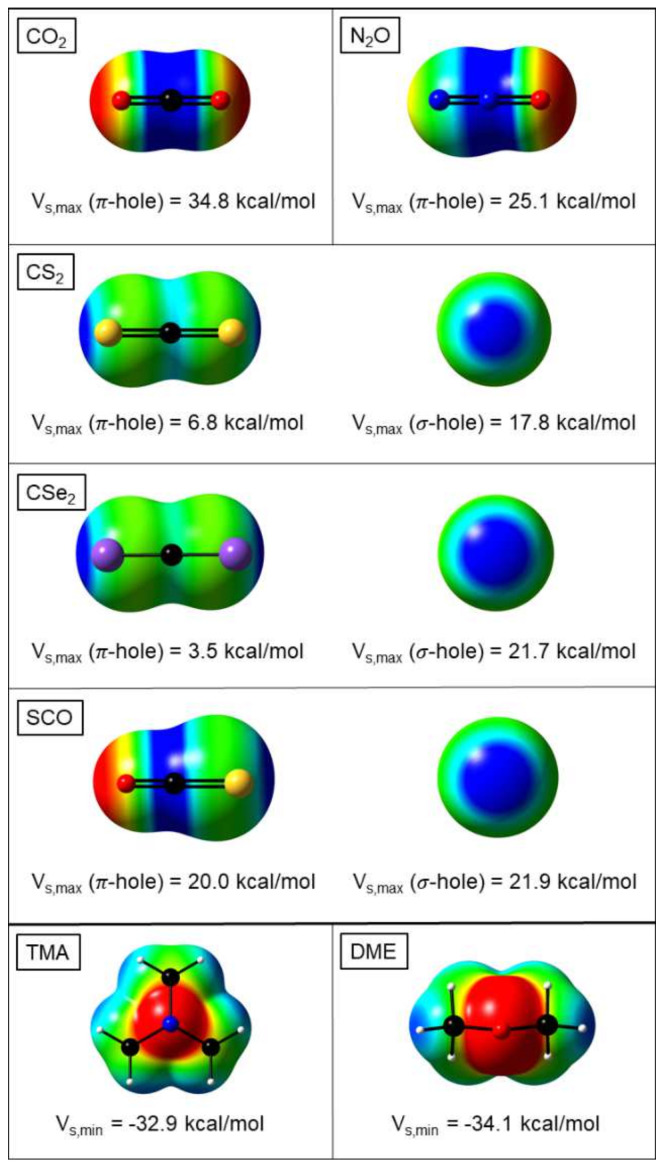
MEP contours, computed with MP2/aug-cc-pVTZ, for the Lewis acids and bases considered. Identical isovalues (ρ(**r**) = 0.01 a.u.) were chosen for all systems. Color-code: red indicates negative charge density and blue indicates positive charge density. V_s,max_ (for the acids) and V_s,min_ (for the bases) values are indicated, in the former case both for the π-hole and the σ-hole (if present). All values in kcal mol^−1^.

In Figure 8, we depict the MEP for CO_2_, CS_2_, CSe_2_, N_2_O, and OCS. Although MEP plots for these molecules have already been published [56,97,100], we preferred to recalculate all of them at the same level of theory, also providing the opportunity for a unique visualization. For CO_2_, CS_2_, N_2_O, and OCS, the overall shape is in agreement with the literature data. To put it now in a more quantitative context using the V_s,max_ values, the series CO_2_, N_2_O, OCS, CS_2_, and CSe_2_ shows a decreasing value of this V_s,max_ for the π-hole, which is very strong in CO_2_, promoting a tetrel-bond and with absence of a σ- hole, thus preventing a chalcogen bond, and then slightly decreases via N_2_O, where the σ-hole is still absent, to OCS, where the V_s,max_ for the σ-hole is slightly higher than for the π-hole (see also [102]). These last findings are in agreement with the elusive tetrel bonding for N_2_O and the discussion above for SCO, where the small difference in V_s,max_ might be compatible with an equilibrium tetrel-chalcogen bond, where secondary effects shift it to the tetrel side, preventing the experimental detection of the chalcogen bond. In line with the quadrupole moment considerations in the previous section, the V_s,max_ value for the π-hole in CS_2_ further decreases and eliminates tetrel bonding as compared with chalcogen bonding, in line with the aforementioned recent experimental microwave studies by Legon et al. on the CS_2_···NH_3_ complex displaying a geometry in line with a chalcogen bond [97] and an extensive series of quantum-chemical calculations on CS_2_ complexes, all pointing to chalcogen bonds [57]. Moving on to CSe_2_, the tetrel-bond forming capacity obviously further reduces in line with the increasing positive value of the quadrupole moment.

Finally, it should be noticed that the MEP surfaces for the Lewis bases (TMA and DME) show similar V_s,min_ values, which agrees with the overall small interaction energy differences between these two, as discussed above.

### 4.3. Energy Decomposition Analysis

We continue our discussion with a quantum-mechanical energy decomposition analysis (EDA) of the Lewis acid–Lewis base interaction energy to ascertain that electrostatics is indeed the most dominant interaction component, thereby substantiating our argumentation of the classical quadrupole–dipole model. The EDA was carried out using PBE0-D3BJ, in combination with the TZ2P basis set specific to the ADF software, instead of the better performing M06-2X-D3 functional, as the (almost) non-empirical PBE0 is dispersion-correction free (see benchmark in ESI, Table S3), a key characteristic when applying the energy decomposition. Despite the overall larger deviations from the experimentally determined complexation energies compared with M06-2X-D3, PBE0-D3BJ correlates very well with the experimental values, with an R^2^ value of 0.88, again excluding the TMA···N_2_O complex, which is even slightly better than for M06-2X-D3 and CCSD(T). The correlation plot is provided in the ESI (Figure S1).

From the EDA energy values in Table 5, it is seen that the electrostatic term has by far the most attractive contribution in the interaction with an average percentual contribution of 62%, justifying the use of the electrostatic model in Section 4.1 as a primer to understand the observed and calculated sequences. Focusing on the tetrel and pnictogen bonded complexes within the series for one type of Lewis base (DME or TMA), it is seen that the two OCS tetrel complexes diverge somewhat from the overall agreement in terms of the electrostatics′ relative contribution to the interaction energy. For the DME perpendicular complexes, the sequence in absolute Δ*V*_elst_ values is in line with the quadrupole value sequence, be it that the difference between N_2_O and OCS does not reflect the large difference in quadrupole moment. However, in the case of TMA, N_2_O and OCS are inverted, with TMA···OCS displaying more stabilizing electrostatic interactions than TMA···N_2_O. As conjectured above, one might ascribe this effect to the fact that OCS has a much larger dipole moment than N_2_O, so that, in this complex, the “reverse” dipole–quadrupole moment interaction may also be at stake, and thereby strengthen the overall interaction of OCS as compared with N_2_O. The absolute contribution of the dispersion term is systematically higher in the TMA complexes (as expected in view of the larger volume/number of electrons of this base), but is also counteracted by a significantly larger destabilizing contribution of the Pauli repulsion term. The orbital interaction term is systematically smaller for the pnictogen bond. Note also that the orbital interactions play a more significant role, both in the absolute and in the relative sense, for the chalcogen bond compared with the tetrel bond with OCS, especially in the case of TMA, probably owing to the better alignment (end-on) of the interacting species, but resulting in a less attractive dispersion component. Lastly, the strongly interacting tetrel complexes with CO_2_ and the extensively dispersion-stabilized TMA···OCS tetrel complex are accompanied by a larger strain energy. On a final note, the PBE0-D3BJ density approximation seems to cause an overstabilization of the chalcogen-bonded complexes, compared with the OCS tetrel bonds, which is in contrast to the CCSD(T) and M06-2X-D3 results and contradicts the experimental observations.

All in all, the energy decomposition analysis shows that the larger part of the interaction energy can be ascribed to the electrostatic term, justifying and underpinning the “classical” electrostatic reasoning presented in the quadrupole–dipole moment model presented in Section 4.1.

### 4.4. Bonding Analysis

**Figure 9:**
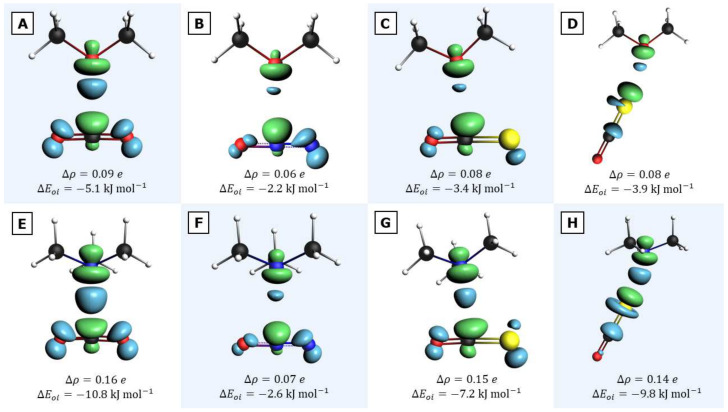
Main component of the NOCV analysis: contours of deformation density distribution Δρ. Electron depletion is indicated by the green-colored regions; electron accumulation is represented by the blue-colored regions. (**A**) DME···CO_2_ (tetrel); (**B**) DME···N_2_O (pnictogen); (**C**) DME···OCS (tetrel); (**D**) DME···OCS (chalcogen); (**E**) TMA···CO_2_ (tetrel); (**F**) TMA···N_2_O (pnictogen); (**G**) TMA···OCS (tetrel); (**H**) TMA···OCS (chalcogen).

A final quantum-chemical analysis is applied to elucidate the impact of charge transfer on the stabilization of the different complexes. To this end, a natural orbital for chemical valence (NOCV) analysis is carried out, in which the charge density reorganization resulting from the interacting orbitals of the two monomers is further decomposed into so-called NOCV orbital contributions. The results of the NOCV analysis are summarized in Table 6 and Figure 9. It turns out that, in all cases, only one contribution is dominant, as shown by the percentage of orbital interactions involved (40–72%). The charge distribution rearrangement Δρ upon complexation ranges from a mere 0.06 a.u. for the pnictogen bonds to a more substantial 0.14–0.16 a.u. for the tetrel and chalcogen bonds with TMA. These latter values are in agreement with a recent study on the 6-OTF_3_-fulvene (T = Si, Ge) and NH_3_ tetrel bonds [104]. At least in part, some of these Δρ values suggest possible charge transfer from the O or N lone-pair(s) region to the central tetrel or pnictogen atom, with a spill-over effect to the outer atoms of the triatomic molecule, and to the sulfur atom in the OCS chalcogen bond with further delocalization over the neighboring carbon atom, as presented by the density deformation contours in Figure 9. Note that the NOCV data nicely indicate that, for TMA, the Δρ value is in nearly all cases appreciably larger than for DME, in line with the above discussed higher basicity of TMA. Furthermore, the associated charge-transfer orbital interaction energies for the TMA complexes, except for the pnictogen bond, take up a larger proportion of the total orbital interaction term. These findings can thus rationalize the markedly larger complexation energies for TMA than predicted by the quadrupole–dipole model.

## 5. Conclusions

Analysis of infrared experimental frequency and intensity data of cryogenic solutions in liquid Ar leads to an unambiguous identification of perpendicular tetrel and pnictogen bonding of three linear triatomics, CO_2_, OCS, and N_2_O, with two Lewis bases, DME and TMA. In particular, the lifting of the degeneracy of the ν_2_ bending mode with concomitant and coupled red and blue shift offers most convincing evidence. The complexation enthalpies, obtained by Van ′t Hoff plots, are the first obtained for this type of complex and, after conversion to complexation energies, show the expected order of magnitude when compared with experimental and theoretical literature data on other hydrogen bond congeners such as halogen and chalcogen bonds. Excellent agreement is obtained between experimental and theoretical (CCSD(T) and M06-2X DFA level) values, certainly in view of the order of magnitude of the values (lower than 20 kJ mol^−1^ and with differences in the sequence of sometimes only a few kJ mol^−1^) and experimental errors. Although theoretically predicted, the chalcogen bond of OCS could not be observed, probably most likely owing to entropic solvation effects. The observed and calculated frequencies are successfully explained using a classical dipole–quadrupole model in which the magnitude and the sign of the quadrupole moment of the triatomics are preponderant. Its use is justified and validated later on by a study of the molecular electrostatic potential focusing on the V_s,max_ values of both σ- and π-holes and, afterwards, in a detailed energy decomposition analysis where the electrostatic interaction term invariably and largely dominates. The orbital interaction term on the other hand mainly accounts for the charge transfer contribution between the oxygen and nitrogen lone pair regions of the triatomics to the tetrel or pnictogen atom with a spill-over effect to the terminal atoms of the triatomics. In view of the much higher basicity of TMA as compared with DME, these charge shifts are appreciably larger in the former case. In summary, the present study offers unambiguous experimental evidence for the existence of perpendicular tetrel and pnictogen bond formation of the linear triatomic Lewis acids. These findings together with quantitative information on their stability are backed by extensive theoretical arguments at different levels of sophistication.

## Figures and Tables

**Table 1:** Solution and vapor phase complexation enthalpies and complexation energies, in kJ mol^−1^, derived for the different studied complexes with dimethyl ether (DME) and trimethyl amine (TMA). For completeness, the values for the complex of dimethyl ether with CO_2_, already reported in [59,60], are also given ^a^.

Lewis Base	Lewis Acid	Bond Type	$\Delta H_{LAr}^{0}$	$\Delta H_{vapor}^{0}$	$\Delta E_{exp}$
DME	CO_2_	tetrel	−8.0(3)	−14.4(8)	−16.6(8)
DME	N_2_O	pnictogen	−8.0(15)	−13.0(17)	−15.3(17)
DME	OCS	tetrel	−6.5(11)	−11.7(12)	−13.6(12)
TMA	CO_2_	tetrel	−11.2(3)	−17.7(8)	−19.8(8)
TMA	N_2_O	pnictogen	−5.5(8)	−8.3(11)	−10.3(11)
TMA	OCS	tetrel	−4.2(3)	−12.4(9)	−14.1(9)

^a^ For the complexes of trimethyl amine with CO_2_, Van ′t Hoff plots were obtained for both ^12^CO_2_ and ^13^CO_2_. The values reported are the weighted average of the data for both isotopomers.

**Table 2:** Theoretical complexation energies and comparison with experimental complexation energies, given in kJ mol^−1^. MUE = mean unsigned error. RMSE = root mean square error. MAX = maximal error.

Complex	Bond Type	$\Delta E_{exp}$	MP2/aug-cc-pVTZ ^a^	CCSD(T)/CBS ^b^	M06-2X-D3/cc-pVTZ ^a^
DME···CO_2_	tetrel	−16.7	−15.3	−17.1	−18.0
DME···N_2_O	pnictogen	−15.3	−14.6	−13.4	−13.6
DME···OCS	tetrel	−13.6	−11.2	−12.1	−11.9
DME···OCS	chalcogen	-	−10.3	−10.4	−9.0
TMA···CO_2_	tetrel	−19.9	−16.9	−17.7	−19.9
TMA···N_2_O	pnictogen	−10.3	−15.2	−13.3	−14.9
TMA···OCS	tetrel	−14.2	−13.9	−12.9	−14.5
TMA···OCS	chalcogen	-	−13.6	−12.9	−12.2
**MUE ^c^**			2.1 (1.5)	1.7 (1.5)	1.6 (1.0)
**RMSE ^c^**			2.6 (1.8)	1.9 (1.6)	2.2 (1.2)
**MAX ^c^**			4.9 (3.0)	3.0 (2.2)	4.6 (1.7)

^a^ For the MP2/aug-ccc-pVTZ and M06-2X-D3/cc-pVTZ complexation energies, BSSE is corrected using the Counterpoise method. ^b^ The CCSD(T) energies are extrapolated to the complete basis set limit using the FPA-QZ method (see ESI, Tables S1 and S2). ^c^ The data for the pnictogen bonding complex TMA···N_2_O are not included in the error statistics in parentheses.

**Table 3:** Theoretical (MP2/aug-cc-pVTZ) and experimental dipole (μ) and quadrupole (Q) moments (values in Debye for the dipole moment (1 a.u. = 2.541D) and 10^−26^ esu cm^2^ for the quadrupole moment (1 a.u. = 1.346 × 10^−26^ esu cm^2^)) for the Lewis acids and bases used in this study.

Monomer	μ		Q	
	Calculated	Experimental	Calculated	Experimental
TMA	0.664	0.612 [89]		
DME	1.345	1.30 [89]		
CO_2_	0	0	−4.14	−4.28 [90]
CS_2_	0	0	+3.18	+3.45 [91]
CSe_2_	0	0	+5.00	-
N_2_O	0.172	0.167 ^a^ [89]	−3.61	−3.30 [92]
OCS	0.710	0.715 ^b^ [93]	−0.77	−0.78 [94]

^a^ Pointing from N to O. ^b^ Pointing from O to S.

**Table 4:** MP2/aug-cc-pVTZ) geometrical parameters to determine the shape (α for T shape, β for quasi-end-on, in °) and the Lewis acid–Lewis base non-covalent bond distance (d_A2__···__B_, d_A3__···__B_ in Å).

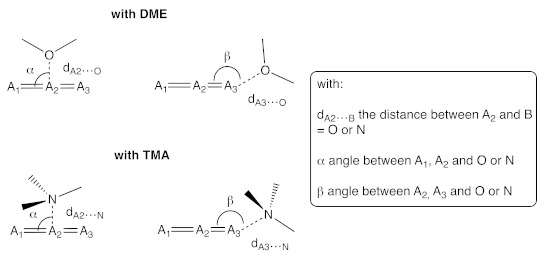						
Complex	Bond Type	Atom Type	d_A2···B_	α	d_A3···B_	β
DME···CO_2_	tetrel	A_1_ = O, A_2_ = C, A_3_ = O, B = O	2.61	91.3	-	-
DME···N_2_O	pnictogen	A_1_ = N, A_2_ = N, A_3_ = O, B = O	2.69	92.1	-	-
DME···OCS	tetrel	A_1_ = O, A_2_ = C, A_3_ = S, B = O	2.75	85.2	-	-
DME···OCS	chalcogen	A_1_ = O, A_2_ = C, A_3_ = S, B = O	-	-	2.92	170.9
TMA···CO_2_	tetrel	A_1_ = O, A_2_ = C, A_3_ = O, B = N	2.66	92.3	-	-
TMA···N_2_O	pnictogen	A_1_ = N, A_2_ = N, A_3_ = O, B = N	2.82	92.4	-	-
TMA···OCS	tetrel	A_1_ = O, A_2_ = C, A_3_ = S, B = N	2.81	86.9	-	-
TMA···OCS	chalcogen	A_1_ = O, A_2_ = C, A_3_ = S, B = N	-	-	2.93	170.2

**Table 5:** Energy decomposition analysis (EDA) of the interaction energy of all eight complexes (cf Equation (5)). All terms are given in kJ mol^−1^. The percentages written in parentheses represent the relative contribution of the orbital interaction energy, the electrostatic energy, and the dispersion energy with respect to the total stabilization component of the interaction energy.

Complex	Bond Type	Δ*E*_complex_	Δ*E*_strain_	Δ*E*_int_	Δ*E*_Pauli_	Δ*V*_elst_	Δ*E*_oi_	*E*_disp_
DME···CO_2_	tetrel	−16.0	0.6	−16.7	26.3	−29.2 (68)	−8.5 (20)	−5.3 (12)
DME···N_2_O	pnictogen	−12.0	0.1	−12.1	20.6	−22.5 (69)	−5.1 (15)	−5.1 (16)
DME···OCS	tetrel	−9.7	0.3	−10.0	23.1	−19.7 (59)	−6.8 (20)	−6.7 (20)
DME···OCS	chalcogen	−9.9	0.1	−10.0	17.2	−15.3 (56)	−7.3 (27)	−4.6 (17)
TMA···CO_2_	tetrel	−19.6	1.7	−21.2	45.4	−42.9 (64)	−15.0 (23)	−8.7 (13)
TMA···N_2_O	pnictogen	−12.9	0.1	−13.0	29.4	−27.5 (65)	−6.6 (15)	−8.3 (20)
TMA···OCS	tetrel	−12.6	0.7	−13.3	40.5	−30.7 (57)	−11.9 (22)	−11.3 (21)
TMA···OCS	chalcogen	−14.1	0.2	−14.2	35.3	−27.5 (56)	−14.2 (29)	−7.8 (16)

**Table 6:** NOCV analysis of the bonding pattern in all eight complexes. Overall deformation density (Δρ) and the absolute and relative contribution of the largest component in the NOCV analysis are given. Energy values are in kJ mol^−1^.

Complex	Bond Type	Δρ	Δ*E*_oi,NOCV_	Δ*E*_oi,NOCV_/Δ*E*_oi,total_ (in %)
DME···CO_2_	tetrel	0.09	−5.1	60
DME···N_2_O	pnictogen	0.06	−2.2	43
DME···OCS	tetrel	0.08	−3.4	50
DME···OCS	chalcogen	0.08	−3.9	53
TMA···CO_2_	tetrel	0.16	−10.8	72
TMA···N_2_O	pnictogen	0.07	−2.6	40
TMA···OCS	tetrel	0.15	−7.2	60
TMA···OCS	chalcogen	0.14	−9.8	69

## Data Availability

Data supporting reported results can be found in the ESI.

## Supplementary Materials

The supplementary materials include CCSD(T)/CBS Results Using Feller and Schwartz Extrapolation: Tables S1 and S2, Density Functional Approximation Benchmark of the Complexation Energies at 0K and Excluding Zero-Point Energies: Table S3, Complexation Energy Correlation Plots of PBE0-D3BJ/cc-pVTZ with Experiment and with PBE0-D3BJ/TZ2P: Figure S1, Cartesian Coordinates of the Isolated Lewis Acids and Bases and their Complexes (at MP2/aug-cc-pVTZ level), Observed Vibrational Frequencies for the 1:1 Complexes and the Shifts Induced by Complexation: Tables S4−S9, MP2/6-311++G(d,p) Calculated Harmonic Frequencies and Infrared Intensities with the Frequency Shifts Induced by the Complexation: Tables S10−S17.
