# Supplementary material for: A Combined Experimental/Quantum-Chemical Study of Tetrel, Pnictogen, and Chalcogen Bonds of Linear Triatomic Molecules
Source: Molecules. 2021 Nov 9;26(22):6767. doi: 10.3390/molecules26226767 (PMC8623034; doi:10.3390/molecules26226767)
Supplement: Supplementary file 1 [file molecules-26-06767-s001.zip › molecules-1452554-supplementary.pdf]

SUPPORTING INFORMATION

for

A Combined Experimental / Quantum-Chemical Study of Tetrel, Pnictogen and Chalcogen Bonds of Linear Triatomic Molecules.

Freija De Vleeschouwer[1], Frank De Proft[1,*], Özge Ergün[1], Wouter Herrebout[2,*] and Paul Geerlings[1,*]

1 Research Group of General Chemistry (ALGC), Vrije Universiteit Brussel (VUB), Pleinlaan 2, B-1050 Brussels, Belgium

2 Molecular Spectroscopy Research Group, Department of Chemistry, University of Antwerp (UA) , Groenenborgerlaan 171 , B-2020 Antwerp, Belgium

Table of Contents

1. CCSD(T)/CBS RESULTS USING FELLER AND SCHWARTZ EXTRAPOLATION S3

2. DENSITY FUNCTIONAL APPROXIMATION BENCHMARK OF THE COMPLEXATION ENERGIES AT 0K AND EXCLUDING ZERO-POINT ENERGIES S4

3. COMPLEXATION ENERGY CORRELATION PLOTS OF PBE0-D3BJ/CC-PVTZ WITH EXPERIMENT AND WITH PBE0-D3BJ/TZ2P. S5

4. CARTESIAN COORDINATES OF THE ISOLATED LEWIS ACIDS AND BASES AND THEIR COMPLEXES (AT MP2/AUG-CC-PVTZ LEVEL) S6

5. OBSERVED VIBRATIONAL FREQUENCIES FOR THE 1:1 COMPLEXES AND THE SHIFTS INDUCED BY COMPLEXATION (TABLES S4-S9)... S10

6. MP2/6-311++G(D,P) CALCULATED HARMONIC FREQUENCIES AND INFRARED INTENSITIES WITH THE FREQUENCY SHIFTS INDUCED BY THE COMPLEXATION (TABLES S10-S17). S18

1. CCSD(T)/CBS Results Using Feller and Schwartz Extrapolation

Table S1. CCSD(T) energies for the cc-pVDZ, cc-pVTZ and cc-pVQZ basis sets, Feller three-point extrapolation of the Hartree-Fock energies $E_{\mathrm{HF}}(\infty)$ and CCSD(T)/CBS energies $S_{\mathrm{CCSD(T)}}(\infty)$ using the Schwartz three-point extrapolation of the correlation energy, defined as CCSD(T) – HF, for all monomers, given in Hartree.

**CO_2**	**DZ**	**TZ**	**QZ**	$\boldsymbol{E_{HF}(\infty)}$	$\boldsymbol{S_{\mathrm{CCSD(T)}}(\infty)}$
HF	-187.65020	-187.70619	-187.72056	-187.72553	
CCSD(T)	-188.13293	-188.30827	-188.36802		-188.41452
**N_2O**	**DZ**	**TZ**	**QZ**	$\boldsymbol{E_{HF}(\infty)}$	$\boldsymbol{S_{\mathrm{CCSD(T)}}(\infty)}$
HF	-183.69094	-183.74377	-183.75705	-183.76151	
CCSD(T)	-184.22452	-184.39359	-184.45077		-184.49526
**OCS**	**DZ**	**TZ**	**QZ**	$\boldsymbol{E_{HF}(\infty)}$	$\boldsymbol{S_{\mathrm{CCSD(T)}}(\infty)}$
HF	-510.28977	-510.33817	-510.35107	-510.35575	
CCSD(T)	-510.72674	-510.88502	-510.93889		-510.98066
**DME**	**DZ**	**TZ**	**QZ**	$\boldsymbol{E_{HF}(\infty)}$	$\boldsymbol{S_{\mathrm{CCSD(T)}}(\infty)}$
HF	-154.07571	-154.12635	-154.1379	-154.14123	
CCSD(T)	-154.55369	-154.72453	-154.77745		-154.81647
**TMA**	**DZ**	**TZ**	**QZ**	$\boldsymbol{E_{HF}(\infty)}$	$\boldsymbol{S_{\mathrm{CCSD(T)}}(\infty)}$
HF	-173.28170	-173.33329	-173.34531	-173.34896	
CCSD(T)	-173.89413	-174.08668	-174.14630		-174.19033

Table S2. CCSD(T) energies for the cc-pVDZ, cc-pVTZ and cc-pVQZ basis sets, Feller three-point extrapolation of the Hartree-Fock energies $E_{\mathrm{HF}}(\infty)$ and CCSD(T)/CBS energies $S_{\mathrm{CCSD(T)}}(\infty)$ using the Schwartz three-point extrapolation of the correlation energy, defined as CCSD(T) – HF, for all complexes, given in Hartree.

**DME⋯CO_2**	**DZ**	**TZ**	**QZ**	$\boldsymbol{E_{HF}(\infty)}$	$\boldsymbol{S_{\mathrm{CCSD(T)}}(\infty)}$
HF	-341.73138	-341.83566	-341.86092	-341.86899	
CCSD(T)	-342.69435	-343.03949	-343.15183		-343.23723
**DME⋯N_2O**	**DZ**	**TZ**	**QZ**	$\boldsymbol{E_{HF}(\infty)}$	$\boldsymbol{S_{\mathrm{CCSD(T)}}(\infty)}$
HF	-337.76997	-337.87141	-337.89556	-337.90311	
CCSD(T)	-338.78483	-339.12407	-339.23388		-339.31732
**DME⋯OCS (tetrel)**	**DZ**	**TZ**	**QZ**	$\boldsymbol{E_{HF}(\infty)}$	$\boldsymbol{S_{\mathrm{CCSD(T)}}(\infty)}$
HF	-664.36788	-664.4648	-664.48855	-664.49628	
CCSD(T)	-665.28610	-665.61452	-665.72112		-665.80193
**DME⋯OCS (chalcogen)**	**DZ**	**TZ**	**QZ**	$\boldsymbol{E_{HF}(\infty)}$	$\boldsymbol{S_{\mathrm{CCSD(T)}}(\infty)}$
HF	-664.36675	-664.46436	-664.48843	-664.49630	
CCSD(T)	-665.28523	-665.61398	-665.72074		-665.80155
**TMA⋯CO_2**	**DZ**	**TZ**	**QZ**	$\boldsymbol{E_{HF}(\infty)}$	$\boldsymbol{S_{\mathrm{CCSD(T)}}(\infty)}$
HF	-360.93555	-361.04042	-361.06595	-361.07417	
CCSD(T)	-362.03608	-362.40299	-362.52169		-362.61176
**TMA⋯N_2O**	**DZ**	**TZ**	**QZ**	$\boldsymbol{E_{HF}(\infty)}$	$\boldsymbol{S_{\mathrm{CCSD(T)}}(\infty)}$
HF	-356.97411	-357.07627	-357.10068	-357.10834	
CCSD(T)	-358.12614	-358.48741	-358.60359		-358.69167
**TMA⋯OCS (tetrel)**	**DZ**	**TZ**	**QZ**	$\boldsymbol{E_{HF}(\infty)}$	$\boldsymbol{S_{\mathrm{CCSD(T)}}(\infty)}$
HF	-683.57173	-683.66916	-683.69320	-683.70107	
CCSD(T)	-684.62795	-684.97837	-685.09139		-685.17689
**TMA⋯OCS (chalcogen)**	**DZ**	**TZ**	**QZ**	$\boldsymbol{E_{HF}(\infty)}$	$\boldsymbol{S_{\mathrm{CCSD(T)}}(\infty)}$
HF	-683.57163	-683.66972	-683.69415	-683.70226	
CCSD(T)	-684.62731	-684.97788	-685.09122		-685.17693

2. Density Functional Approximation Benchmark of the Complexation Energies at 0K and Excluding Zero-Point Energies

Table S3. Theoretical complexation energies for a set of DFT functionals and basis sets, and comparison with experimental complexation energies, given in kJ mol^{-1}. For all levels of theory, the Counterpoise method was used to correct for the basis set superposition error. MUE = mean unsigned error. RMSE = root mean square error. MAX: maximal error.

Complex	Bond type	ΔE_{exp}	ωB97X-D		B97X-D		M11		M06-2X-D3		PBE0	PBE0-D3BJ
			TZ	aug-TZ	TZ	aug-TZ	TZ	aug-TZ	TZ	aug-TZ	TZ	TZ
DME···CO_2	tetrel	-16.6(8)	-12.3	-12.7	-9.9	-10.5	-15.5	-15.2	-18.0	-18.5	-8.9	-14.2
DME···N_2O	pnictogen	-15.3(17)	-8.6	-8.7	-6.6	-7.0	-11.8	-11.2	-13.6	-13.9	-5.4	-10.5
DME···OCS	tetrel	-13.6(12)	-7.3	-7.4	-6.4	-6.7	-9.6	-9.0	-11.9	-12.1	-2.5	-9.2
DME···OCS	chalcogen	–	-5.3	-5.6	-6.7	-7.0	-7.0	-6.8	-9.0	-9.3	-3.7	-8.2
TMA···CO_2	tetrel	-19.8(8)	-14.8	-15.4	-15.1	-16.0	-16.4	-16.1	-19.9	-20.7	-7.9	-16.6
TMA···N_2O	pnictogen	-10.3(11)	-9.6	-9.7	-8.3	-8.9	-12.7	-11.9	-14.9	-15.3	-2.4	-10.7
TMA···OCS	tetrel	-14.1(9)	-9.7	-9.9	-11.6	-12.1	-11.9	-11.3	-14.5	-15.0	-0.5	-11.8
TMA···OCS	chalcogen	–	-7.9	-8.1	-12.0	-12.2	-9.6	-9.3	-12.2	-12.6	-4.1	-11.9
		**MUE**[a]	*4.6 (5.4)*	*4.4 (5.1)*	*5.3 (6.0)*	*4.8 (5.5)*	*2.8 (2.9)*	*3.1 (3.4)*	*1.6 (1.0)*	*1.9 (1.3)*	*10.4 (10.9)*	*3.0 (3.5)*
		**RMSE**[a]	*5.0 (5.5)*	*4.8 (5.2)*	*5.9 (6.4)*	*5.4 (5.9)*	*3.0 (3.1)*	*3.3 (3.5)*	*2.2 (1.2)*	*2.4 (1.3)*	*10.6 (11.1)*	*3.3 (3.6)*
		**MAX**[a]	*6.7 (6.7)*	*6.6 (6.6)*	*8.7 (8.7)*	*8.3 (8.3)*	*4.0 (4.0)*	*4.6 (4.6)*	*4.6 (1.7)*	*5.0 (1.8)*	*13.7 (13.7)*	*4.8 (4.8)*

[a] The data for the pnictogen bonding complex TMA···N_2O are not included in the error statistics in brackets.

3. Complexation Energy Correlation Plots of PBE0-D3BJ/cc-pVTZ with Experiment and with PBE0-D3BJ/TZ2P.

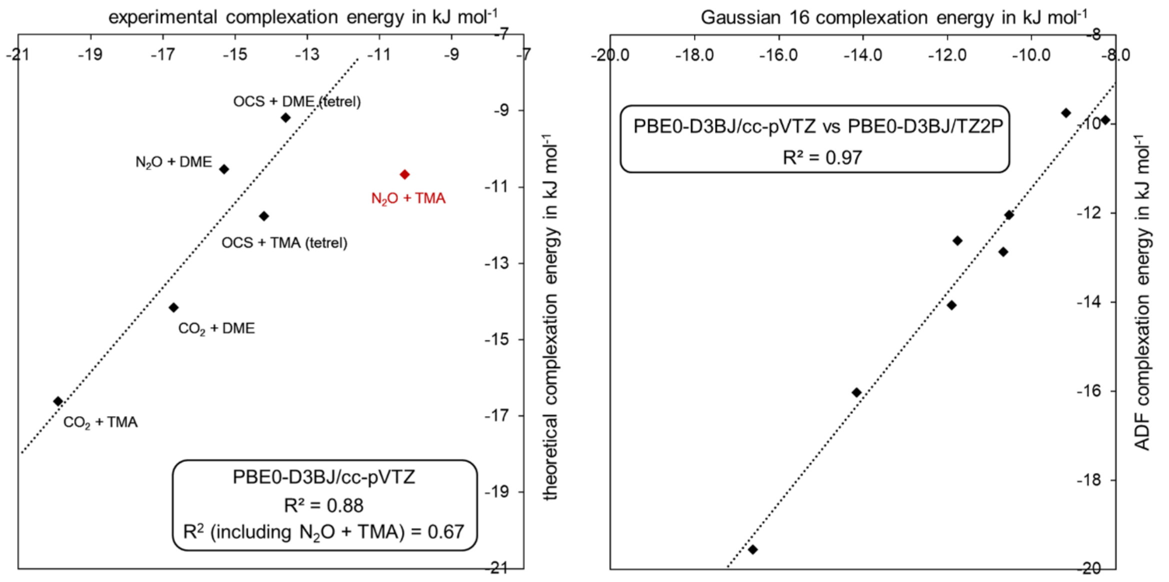

Figure S1. Left: correlation between PBE0-D3BJ/cc-pVTZ and experimental complexation energies. Right: correlation between PBE0-D3BJ/cc-pVTZ (Gaussian 16) and PBE0-D3BJ/TZ2P (ADF) complexation energies. The dashed line represents the linear regression line. Pnictogen complex N2O + TMA is excluded from the linear regression analysis in the left scatter plot.

4. Cartesian Coordinates of the Isolated Lewis Acids and Bases and their Complexes (at MP2/aug-cc-pVTZ level)

CO_2

C	0.00000000	0.00000000	0.00000000
O	0.00000000	0.00000000	1.16566900
O	0.00000000	0.00000000	-1.16566900

N_2O

O	0.00000000	0.00000000	1.11440200
N	0.00000000	0.00000000	-1.21161600
N	0.00000000	0.00000000	-0.06198600

OCS

O	0.00000000	0.00000000	-1.68640900
C	0.00000000	0.00000000	-0.52218500
S	0.00000000	0.00000000	1.03902400

DME

O	0.00000000	0.00000000	0.60001800
C	0.00000000	1.15801100	-0.19907800
H	-0.88701800	1.19625300	-0.83582900
H	0.00000000	2.01428500	0.46605300
H	0.88701800	1.19625300	-0.83582900
C	0.00000000	-1.15801100	-0.19907800
H	0.00000000	-2.01428500	0.46605300
H	-0.88701800	-1.19625300	-0.83582900
H	0.88701800	-1.19625300	-0.83582900

TMA

C	0.00000000	1.36832400	-0.06451800
H	-0.88289600	1.88221600	0.30748300
H	0.88289600	1.88221600	0.30748300
H	0.00000000	1.43142200	-1.16336400
C	-1.18500300	-0.68416200	-0.06451800
H	-1.18859900	-1.70571800	0.30748300
H	-2.07149500	-0.17649800	0.30748300
H	-1.23964800	-0.71571100	-1.16336400
N	0.00000000	0.00000000	0.40093100
C	1.18500300	-0.68416200	-0.06451800
H	2.07149500	-0.17649800	0.30748300
H	1.18859900	-1.70571800	0.30748300
H	1.23964800	-0.71571100	-1.16336400

DME···CO_2

O	0.00000000	0.00000000	0.88218800
C	0.00000000	1.16502000	1.67346200
H	0.88810000	1.20628200	2.30760700
H	0.00000000	2.01157500	0.99566000
H	-0.88810000	1.20628200	2.30760700
C	0.00000000	-1.16502000	1.67346200
H	0.00000000	-2.01157500	0.99566000
H	0.88810000	-1.20628200	2.30760700
H	-0.88810000	-1.20628200	2.30760700
C	0.00000000	0.00000000	-1.72405900
O	0.00000000	1.16548300	-1.75102800
O	0.00000000	-1.16548300	-1.75102800

DME···N_2O

O	0.00000000	0.90840900	0.00000000
C	1.15851200	1.70856700	0.00000000
H	1.19588200	2.34371200	0.88782300
H	2.01097800	1.03808800	0.00000000
H	1.19588200	2.34371200	-0.88782300
C	-1.16670100	1.69699900	0.00000000
H	-2.01320800	1.01889100	0.00000000
H	-1.21012600	2.33153300	0.88804800
H	-1.21012600	2.33153300	-0.88804800
O	1.11701500	-1.76501700	0.00000000
N	-1.20684600	-1.79353500	0.00000000
N	-0.05833500	-1.77618000	0.00000000

DME···OCS (tetrel)

O	0.00000000	1.26408500	0.00000000
C	1.25695100	1.89618900	0.00000000
H	1.38293500	2.51956600	0.88824300
H	2.00853400	1.11379700	0.00000000
H	1.38293500	2.51956600	-0.88824300
C	-1.04880200	2.20346600	0.00000000
H	-1.97829700	1.64456300	0.00000000
H	-1.00639500	2.83818200	0.88802400
H	-1.00639500	2.83818200	-0.88802400
O	-1.67639900	-1.10070500	0.00000000
C	-0.56141600	-1.43268000	0.00000000
S	0.92171700	-1.92392200	0.00000000

DME···OCS (chalcogen)

O	0.49984700	-1.79255700	0.00000000
C	-0.78666400	-2.36550800	0.00000000
H	-0.94070000	-2.98199600	0.88832400
H	-1.50322000	-1.55089800	0.00000000
H	-0.94070000	-2.98199600	-0.88832400
C	1.49720800	-2.78728200	0.00000000
H	2.45769200	-2.28407200	0.00000000
H	1.41960800	-3.41849500	0.88785200
H	1.41960800	-3.41849500	-0.88785200
O	-0.88965100	3.65977600	0.00000000
C	-0.50952000	2.55734200	0.00000000
S	0.00000000	1.07943100	0.00000000

TMA···CO_2

C	0.37313000	-1.49512900	1.19144900
H	1.45974000	-1.53000200	1.18754500
H	0.04908800	-0.94209400	2.06970000
H	-0.00909100	-2.52352800	1.26212600
C	0.37313000	-1.49512900	-1.19144900
H	0.04908800	-0.94209400	-2.06970000
H	1.45974000	-1.53000200	-1.18754500
H	-0.00909100	-2.52352800	-1.26212600
O	0.37313000	1.84589400	-1.16606700
N	-0.09545200	-0.82292000	0.00000000
C	-1.53976700	-0.74616900	0.00000000
H	-1.87574500	-0.20923800	0.88472900
H	-1.87574500	-0.20923800	-0.88472900
H	-2.00636700	-1.74176100	0.00000000
C	0.36958300	1.79936400	0.00000000
O	0.37313000	1.84589400	1.16606700

TMA···N_2O

C	-1.61665100	-1.13486700	-0.59234700
H	-1.65200100	-1.07054900	-1.67712100
H	-1.11090700	-2.05747000	-0.31710000
H	-2.64815200	-1.17420400	-0.21186000
C	-1.50428100	1.23720000	-0.45881700
H	-0.91777600	2.07101300	-0.08011200
H	-1.53411500	1.30120300	-1.54373600
H	-2.53153100	1.32851300	-0.07578200
O	1.91352400	1.11015400	-0.11985800
N	-0.88385700	-0.00680300	-0.06232800
C	-0.81027000	-0.09162900	1.37883500
H	-0.32462400	-1.02170500	1.66595800
H	-0.22593300	0.74175700	1.76376400
H	-1.80475800	-0.06140100	1.84791900
N	1.95562900	-1.21403700	-0.14316300
N	1.93234400	-0.06524700	-0.13438000

TMA···OCS (tetrel)

C	-1.53582800	-1.40484700	-0.53201200
H	-1.44861300	-1.42777800	-1.61574900
H	-0.85808000	-2.14736200	-0.11596100
H	-2.56516300	-1.67996700	-0.25812200
C	-2.01314000	0.92708800	-0.62079000
H	-1.68027300	1.90401300	-0.27774900
H	-1.93312100	0.89661800	-1.70481200
H	-3.07065000	0.80054200	-0.34555000
O	1.15673500	1.77035100	-0.09583500
C	1.53274700	0.66849600	-0.08401800
S	2.11283000	-0.78285800	-0.05761800
N	-1.17212100	-0.09572000	-0.04347200
C	-1.24075700	-0.05509100	1.39870200
H	-0.56792800	-0.80107700	1.81653800
H	-0.93241300	0.92741400	1.75031000
H	-2.25619700	-0.25331600	1.77266700

TMA···OCS (chalcogen)

C	-0.43022700	-1.88560300	1.18633200
H	0.16050200	-1.66296200	2.07176500
H	-1.30744300	-1.24198500	1.18600900
H	-0.76660300	-2.93111400	1.24409300
C	1.55486900	-2.43838500	0.00000000
H	2.14722600	-2.21111700	-0.88301500
H	2.14722600	-2.21111700	0.88301500
H	1.32842500	-3.51459800	0.00000000
O	-0.76250100	3.89706000	0.00000000
C	-0.43022700	2.77928400	0.00000000
S	0.01452900	1.27708900	0.00000000
N	0.35523200	-1.63130200	0.00000000
C	-0.43022700	-1.88560300	-1.18633200
H	-1.30744300	-1.24198500	-1.18600900
H	0.16050200	-1.66296200	-2.07176500
H	-0.76660300	-2.93111400	-1.24409300

5. Observed Vibrational Frequencies for the 1:1 Complexes and the Shifts Induced by Complexation (Tables S4-S9).

Table S4. Observed vibrational frequencies, in cm^{-1}, for the 1:1 complex of trimethyl amine with $^{12}CO_2$. For completeness, also the shifts induced by the complexation with the partner molecule are given.

	Assignment	Monomer $\tilde{\nu}$	Complex $\tilde{\nu}$	$\Delta\tilde{\nu}$
$N(CH_3)_3$	$\nu_{12}(e)$	2979.7	2982.1	2.4
	$2(\nu_{20}+\nu_{21})$	2956.7	2957.4	0.7
	$\nu_1(a_1), \nu_{13}(e)$	2947.2	2948.7	1.5
	$2\nu_{17}$	2820.6	2825.8	5.2
	$\nu_2(a_1), \nu_{14}(e)$	2771.0	2776.3	5.3
	$\nu_{15}+\nu_{18}$	2733.7	2733.8	0.1
	$\nu_3+\nu_5$	2637.1	2642.8	5.7
	$\nu_{16}+\nu_5$	2626.0	2631.9	5.9
		2589.0	2595.7	6.7
	$\nu_{15}+\nu_{20}$	2495.5	2497.9	2.4
	$\nu_{18}+\nu_6$	2098.9	2096.0	-2.9
	$2\nu_{20}$	2080.5	2076.4	-4.1
	$\nu_5+\nu_6$	2011.0	2015.6	4.6
	$\nu_{20}+\nu_6$	1867.0	1865.1	-1.9
	$\nu_{20}+\nu_{21}$	1475.9	1475.2	-0.7
	$\nu_{15}(e)$	1468.9	1469.6	0.7
	$\nu_3(a_1)$	1456.1	1455.8	-0.3
	$\nu_{16}(e)$	1440.2	1441.5	1.3
	$\nu_{17}(e)$	1415.8	1424.5	8.7
		1294.2	1288.8	-5.4
	$\nu_{18}(e)$	1274.1	1272.7	-1.4
	$\nu_5(a_1)$	1185.2	1190.5	5.3
	$\nu_{19}(e)$	1099.1	1099.4	0.3
	$\nu_{20}(e)$	1042.1	1039.4	-2.7
	$2\nu_{21}$	852.0	851.0	-1.0
	$\nu_6(a_1)$	828.4	828.2	-0.2
$^{12}CO_2$	$\nu_1+\nu_3$	3704.2	3690.5	-13.7
	$2\nu_2+\nu_3$	3601.6	3606.7	5.1
	$\nu_3(\Sigma_u^+)$	2341.8	2334.3	-7.5
	$\nu_{2a}(\Pi_g)$	664.3	669.9	5.6
	$\nu_{2b}(\Pi_u)$	664.3	628.9	-35.4

Table S5. Observed vibrational frequencies, in cm^{-1}, for for the 1:1 complex of trimethyl amine with $^{13}CO_2$. For completeness, also the shifts induced by the complexation with the partner molecule are given.

		Monomer	Complex	
	Assignment	$\tilde{\nu}$	$\tilde{\nu}$	$\Delta\tilde{\nu}$
$N(CH_3)_3$	$\nu_{12}(e)$	2979.7	2982.2	2.5
	$2(\nu_{20}+\nu_{21})$	2956.7	2957.3	0.6
	$\nu_1(a_1),\nu_{13}(e)$	2947.2	2948.9	1.7
	$2\nu_{17}$	2820.6	2825.6	5.0
	$\nu_2(a_1),\nu_{14}(e)$	2771.0	2776.1	5.1
	$\nu_{15}+\nu_{18}$	2733.7	2733.5	-0.2
		2718.2	2720.4	2.2
	$\nu_3+\nu_5$	2637.1	2641.9	4.8
	$\nu_{16}+\nu_5$	2626.0	2631.9	5.9
	$\nu_{15}+\nu_{20}$	2495.5	2497.3	1.8
	$\nu_{18}+\nu_6$	2098.9	2096.8	-2.1
	$2\nu_{20}$	2080.5	2076.4	-4.1
	$\nu_5+\nu_6$	2011.0	2015.9	4.9
	$\nu_{20}+\nu_6$	1867.0	1865.4	-1.6
		1524.8	1524.2	-0.6
	$\nu_{20}+\nu_{21}$	1475.9	1475.1	-0.8
	$\nu_{15}(e)$	1468.9	1469.5	0.6
	$\nu_3(a_1)$	1456.1	1455.7	-0.4
	$\nu_{16}(e)$	1440.2	1441.4	1.2
	$\nu_{17}(e)$	1415.8	1424.5	8.7
		1294.2	1288.8	-5.4
	$\nu_{18}(e)$	1274.1	1272.2	-1.9
	$\nu_5(a_1)$	1185.2	1190.2	5.0
	$\nu_{19}(e)$	1099.1	1099.2	0.1
	$\nu_{20}(e)$	1042.1	1040.0	-2.1
	$2\nu_{21}$	852.0	851.5	-0.5
	$\nu_6(a_1)$	828.4	828.2	-0.2
$^{13}CO_2$	$2\nu_2+\nu_3$	3623.4	3609.3	-14.1
	$\nu_3(\Sigma_u^+)$	2276.6	2269.4	-7.2
	$\nu_{2a}(\Pi_g)$	645.9	650.4	4.5
	$\nu_{2b}(\Pi_u)$	645.9	611.4	-34.5

Table S6. Observed vibrational frequencies, in cm^{-1}, for the 1:1 complex of dimethyl ether with N_2O. For completeness, also the shifts induced by the complexation with the partner molecule are given.

	Assignment	Monomer $\tilde{\nu}$	Complex $\tilde{\nu}$	$\Delta\tilde{\nu}$
CH_3OCH_3	$\nu_1(a_1), \nu_{12}(b_1)$	2994.0	2994.5	0.5
		2954.3	2954.9	0.6
	$2\nu_3$	2936.6	2937.0	0.4
	$\nu_3 + \nu_{19}$	2932.0	2933.6	1.6
	$\nu_{18}(b_2)$	2919.5	2920.6	1.1
	$2\nu_{14}$	2913.4	2913.6	0.2
	$\nu_{14} + \nu_{19}$	2908.5	2909.5	1.0
	$2\nu_{19}$	2889.4	2892.1	2.7
	$\nu_3 + \nu_{15}$	2883.6	2886.1	2.5
	$\nu_{14} + \nu_{15}$	2869.7	2871.4	1.7
	$\nu_{15} + \nu_{19}$	2867.7	2868.5	0.8
	$\nu_2(a_1), \nu_{13}(b_1)$	2814.6	2816.8	2.2
		2700.0	2702.8	2.8
		2694.3	2695.1	0.8
		2629.4	2630.1	0.7
		2601.3	2602.3	1.0
		2597.2	2592.4	-4.8
		2416.2	2415.6	-0.6
	$2\nu_{16}(a_1)$	2354.5	2355.2	0.7
	$\nu_6 + \nu_{16}$	2095.3	2092.8	-2.5
		2079.0	2072.7	-6.3
	$\nu_6 + \nu_{17}$	2023.1	2020.5	-2.6
	$2\nu_6$	1854.4	1851.8	-2.6
	$\nu_3(a_1)$	1476.9	1477.3	0.4
	$\nu_{14}(b_1)$	1459.2	1459.3	0.1
	$\nu_{19}(b_2)$	1456.9	1457.7	0.8
	$\nu_5(a_1)$	1246.1	1247.5	1.4
	$\nu_{20}(b_2)$	1167.5	1166.4	-1.1
		1093.2	1091.9	-1.3
	$\nu_6(a_1)$	930.7	929.2	-1.5
		920.3	918.7	-1.6
N_2O	$2\nu_1 + 2\nu_2$	3742.5	3743.2	0.7
	$\nu_1 + \nu_3$	3474.6	3486.9	12.3
	$2\nu_2 + \nu_3$	3358.1	3361.9	3.8
	$2\nu_1$	2559.5	2558.8	-0.7
	$2\nu_1(^{15}N_1)$	2531.1	2531.9	0.8
	$\nu_1 + 2\nu_2$	2458.2	2459.6	1.4
	$\nu_3(\Sigma^-)$	2220.2	2227.9	7.7
	$\nu_3\ ^{15}N_2(\Sigma^-)$	2173.5	2181.7	8.2
	$\nu_1 + \nu_2$	1877.9	1870.1	-7.8
	$\nu_1(\Sigma^+)$	1283.2	1286.3	3.1
	$\nu_{2a}(\Pi)$	588.4	590.7	2.3
	$\nu_{2b}(\Pi)$	588.4	584.8	-3.6

Table S7. Observed vibrational frequencies, in cm^{-1}, for the 1:1 complex of trimethyl amine with N_2O. For completeness, also the shifts induced by the complexation with the partner molecule are given.

	Assignment	Monomer $\tilde{\nu}$	Complex $\tilde{\nu}$	$\Delta\tilde{\nu}$
$N(CH_3)_3$	$\nu_{12}(e)$	2979.7	2981.3	1.6
	$2(\nu_{20}+\nu_{21})$	2956.7	2956.3	-0.4
	$\nu_1(a_1), \nu_{13}(e)$	2947.2	2947.3	0.1
	$2\nu_{17}$	2820.6	2823.3	2.7
	$\nu_2(a_1), \nu_{14}(e)$	2771.0	2773.7	2.7
	$\nu_{15}+\nu_{18}$	2733.7	2732.8	-0.9
	$\nu_3+\nu_5$	2637.1	2636.4	-0.7
	$\nu_{16}+\nu_5$	2626.0	2629.1	3.1
		2589.0	2591.9	2.9
	$\nu_{15}+\nu_{20}$	2495.5	2497.5	2.0
	$\nu_{18}+\nu_6$	2098.9	2096.9	-2.0
		2080.5	2077.4	-3.1
	$\nu_5+\nu_6$	2011.0	2013.4	2.4
	$\nu_{20}+\nu_6$	1867.0	1863.8	-3.2
	$\nu_{20}+\nu_{21}$	1475.9	1475.3	-0.6
	$\nu_{15}(e)$	1468.9	1469.2	0.3
	$\nu_3(a_1)$	1456.1	1455.9	-0.2
	$\nu_{16}(e)$	1440.2	1440.9	0.7
	$\nu_{18}(e)$	1274.1	1272.7	-1.4
	$\nu_5(a_1)$	1185.2	1188.0	2.8
	$\nu_{19}(e)$	1099.1	1099.3	0.2
	$\nu_{20}(e)$	1042.1	1040.6	-1.5
	$2\nu_{21}$	852.0	851.5	-0.5
	$\nu_6(a_1)$	828.4	828.2	-0.2
N_2O	$3\nu_1$	3830.9	3835.3	4.4
	$\nu_1+\nu_3$	3474.6	3484.6	10.0
	$2\nu_2+\nu_3$	3358.1	3359.4	1.3
	$\nu_2+\nu_3$	2793.7	2793.0	-0.7
	$2\nu_1$	2559.5	2563.9	4.4
	$\nu_1+2\nu_2$	2458.2	2457.9	-0.3
	$\nu_3(\Sigma^-)$	2220.2	2226.2	6.0
	$\nu_3\ {}^{15}N_1(\Sigma^-)$	2197.7	2197.0	-0.7
	$\nu_3\ {}^{15}N_2(\Sigma^-)$	2173.5	2173.1	-0.4
	$\nu_1+\nu_2$	1877.9	1877.6	-0.3
	$\nu_1(\Sigma^+)$	1283.2	1273.9	-9.3
	$2\nu_2$	1166.8	1166.2	-0.6
	$\nu_{2a}(\Pi)$	588.2	590.9	2.7
	$\nu_{2b}(\Pi)$	588.2	578.0	-10.2

Table S8. Observed vibrational frequencies, in cm^{-1}, for the tetrel bonded 1:1 complex of dimethyl ether with OCS. For completeness, also the shifts induced by the complexation with the partner molecule are given. [a]

	Assignment	Monomer $\tilde{\nu}$	Complex $\tilde{\nu}$	$\Delta\tilde{\nu}$
CH_3OCH_3	$\nu_1(a_1), \nu_{12}(b_1)$	2994.0	2996.8	2.8
		2994.0	2994.2	0.2
	$2\nu_3$	2936.6	2937.2	0.6
	$\nu_{18}(b_2)$	2919.5	2920.5	1.0
	$2\nu_{19}$	2889.4	2890.8	1.4
	$\nu_3 + \nu_{15}$	2883.6	2886.2	2.6
	$\nu_{14} + \nu_{15}$	2869.7	2871.4	1.7
	$\nu_{15} + \nu_{19}$	2867.7	2868.9	1.2
	$\nu_2(a_1), \nu_{13}(b_1)$	2814.6	2816.9	2.3
		2700.0	2702.5	2.5
		2694.3	2693.9	-0.4
		2646.7	2647.7	1.0
		2622.2	2622.7	0.5
		2601.3	2602.4	1.1
		2597.2	2601.2	4.0
		2416.2	2413.2	-3.0
		2195.5	2194.7	-0.8
	$\nu_3(a_1)$	1476.9	1476.1	-0.8
	$\nu_{14}(b_1)$	1459.2	1459.0	-0.2
	$\nu_{19}(b_2)$	1456.9	1456.8	-0.1
	$\nu_{15}(b_1)$	1427.6	1428.0	0.4
	$\nu_5(a_1)$	1246.1	1246.9	0.8
	$\nu_{16}(b_1), \nu_{20}(b_2)$	1173.6	1173.5	-0.1
	$\nu_{17}(b_1)$	1100.1	1099.9	-0.2
		1093.2	1091.8	-1.4
	$\nu_6(a_1)$	930.7	927.4	-3.3
OCS	$\nu_1 + 2\nu_3$	4935.9	4934.6	-1.3
	$4\nu_2 + \nu_3$	4129.6	4129.3	-0.3
	$2\nu_3$	4082.8	4082.5	-0.3
	$2\nu_3(^{18}O)$	4011.7	4014.2	2.5
	$2\nu_3(^{13}C)$	3978.3	3977.9	-0.4
	$\nu_1 + 2\nu_2 + \nu_3$	3927.2	3926.9	-0.3
		3761.4	3761.7	0.3
	$2\nu_1 + \nu_3$	3756.9	3756.7	-0.2
		3703.2	3700.5	-2.7
	$2\nu_2 + \nu_3$	3085.6	3085.3	-0.3
	$\nu_1 + 4\nu_2$	2933.9	2937.1	3.2
	$\nu_1 + \nu_3$	2908.7	2909.0	0.3
	$\nu_1 + \nu_3(^{34}S)$	2897.0	2896.2	-0.8
	$\nu_1 + \nu_3(^{13}C)$	2852.1	2852.0	-0.1
	$\nu_1 + \nu_3(^{18}O)$	2840.1	2838.6	-1.5
	$2\nu_1 + 2\nu_2$	2729.3	2729.6	0.3
		2706.3	2702.2	-4.1
	$\nu_2 + \nu_3$	2565.7	2564.9	-0.8
	$3\nu_1$	2555.3	2555.6	0.3
	$4\nu_2$	2101.8	2100.8	-0.1
		1963.0	1962.8	-0.2
	$\nu_1 + 2\nu_2$	1890.5	1889.6	-0.9
	$\nu_1 + 2\nu_2(^{34}S)$	1878.0	1878.1	0.1
	$\nu_1 + 2\nu_2(^{13}C)$	1855.2	1855.3	0.1
	$2\nu_1$	1710.6	1710.8	0.2

$2\nu_1(^{13}C)$	1701.7	1701.9	0.2
$2\nu_1(^{34}S)$	1688.3	1688.4	0.1
	1529.1	1529.0	-0.1
$\nu_1 + \nu_2$	1371.8	1371.9	0.1
$2\nu_2$	1045.5	1044.7	-0.8
$2\nu_2(^{13}C)$	1014.7	1014.5	-0.2
$\nu_1(\Sigma^+)$	858.8	858.1	-0.7
$\nu_1(^{34}S)(\Sigma^+)$	847.6	847.7	0.1
$\nu_1(^{18}O)(\Sigma^+)$	838.0	837.8	-0.2
$\nu_{2a}(\Pi)$	519.9	526.1	6.2
$\nu_{2b}(\Pi)$	519.9	512.8	-7.1

[a] no spectral features assigned to a chalcogen bonded complex were observed.

Table S9. Observed vibrational frequencies, in cm^{-1}, for the tetrel bonded 1:1 complex of trimethyl amine with OCS. For completeness, also the shifts induced by the complexation with the partner molecule are given. [a]

	Assignment	Monomer $\tilde{\nu}$	Complex $\tilde{\nu}$	$\Delta\tilde{\nu}$
$N(CH_3)_3$	$\nu_{12}(e)$	2979.7	2979.5	-0.2
	$2(\nu_{20}+\nu_{21})$	2956.7	2955.9	-0.8
	$\nu_1(a_1), \nu_{13}(e)$	2947.2	2946.6	-0.6
	$2\nu_{17}$	2820.6	2823.9	3.3
	$\nu_2(a_1), \nu_{14}(e)$	2771.0	2773.6	2.6
	$\nu_{15}+\nu_{18}$	2733.7	2733.1	-0.6
		2718.2	2717.8	-0.4
	$\nu_3+\nu_5$	2637.1	2639.0	1.9
	$\nu_{16}+\nu_5$	2626.0	2629.1	3.1
	$\nu_{15}+\nu_{20}$	2495.5	2496.1	0.6
	$\nu_{20}+\nu_6$	1867.0	1864.6	-2.4
		1524.8	1523.9	-0.9
	$\nu_{20}+\nu_{21}$	1475.9	1474.7	-1.2
	$\nu_{15}(e)$	1468.9	1469.0	0.1
	$\nu_3(a_1)$	1456.1	1455.4	-0.7
	$\nu_{16}(e)$	1440.2	1440.0	-0.2
	$\nu_{17}(e)$	1415.8	1419.8	4.0
		1294.2	1291.0	-3.2
	$\nu_{18}(e)$	1274.1	1272.5	-1.6
	$\nu_5(a_1)$	1185.2	1187.9	2.7
$N(CH_3)_3$	$\nu_{19}(e)$	1099.1	1098.6	-0.5
	$\nu_{20}(e)$	1042.1	1040.4	-1.7
	$\nu_6(a_1)$	828.4	827.3	-1.1
OCS	$\nu_1+2\nu_2$	4935.9	4935.5	-0.4
	$4\nu_3+\nu_2$	4129.6	4128.2	-1.4
	$2\nu_2$	4082.8	4081.9	-0.9
	$2\nu_2(^{18}O)$	4011.7	4010.9	-0.8
	$2\nu_2(^{13}C)$	3978.3	3977.1	-1.2
	$\nu_1+2\nu_3+\nu_2$	3927.2	3926.5	-0.7
		3761.4	3761.2	-0.2
	$2\nu_1+\nu_2$	3756.9	3756.4	-0.5
		3703.2	3692.1	-11.1
	$\nu_2+2\nu_3$	3085.6	3084.8	-0.8
	$\nu_1+\nu_2$	2908.7	2908.4	-0.3
	$\nu_1+\nu_2(^{34}S)$	2897.0	2896.1	-0.9
	$\nu_1+\nu_2(^{13}C)$	2852.1	2851.2	-0.9
	$\nu_2+\nu_3$	2565.7	2565.3	-0.4
		2558.7	2560.9	2.2
	$3\nu_1$	2555.3	2555.7	0.4
	$4\nu_3$	2101.8	2101.5	-0.3
		1963.0	1962.4	-0.6
	$\nu_1+2\nu_3$	1890.5	1890.8	0.3
	$\nu_1+2\nu_3(^{34}S)$	1878.0	1877.9	-0.1
	$\nu_1+2\nu_3(^{13}C)$	1855.2	1855.1	-0.1
	$2\nu_1$	1710.6	1710.8	0.2
	$2\nu_1(^{13}C)$	1701.7	1702.0	0.3
	$2\nu_1(^{34}S)$	1688.3	1688.7	0.4
		1529.1	1529.0	-0.1

$\nu_1 + \nu_3$	1371.8	1372.9	1.1
$2\nu_3$	1045.5	1041.9	-3.6
$2\nu_3\left(^{13}C\right)$	1014.7	1014.0	-0.7
$\nu_1\left(\Sigma^+\right)$	858.8	858.6	-0.2
$\nu_1\left(^{34}S\right)\left(\Sigma^+\right)$	847.6	847.5	-0.1
$\nu_{3a}\left(\Pi\right)$	519.9	528.9	9.0
$\nu_{3b}\left(\Pi\right)$	519.9	502.7	-17.2

[a] no spectral features assigned to a chalcogen bonded complex were observed.

6. MP2/6-311++G(d,p) Calculated Harmonic Frequencies and Infrared Intensities with the Frequency Shifts Induced by the Complexation (Tables S10-S17).

Table S10. MP2/6-311++G(d,p) calculated harmonic vibrational frequencies, in cm^{-1}, and infrared intensities, in km mol^{-1}, obtained for the 1:1 complex of dimethyl ether with N_2O. For completeness, also the shifts induced by the complexation with the partner molecule are given. Where applicable, BSSE corrected derivatives were used.

		Monomer		Complex		
	Assignment ·	$\tilde{\nu}$	Int ·	$\tilde{\nu}$	Int ·	$\Delta\tilde{\nu}$
CH_3OCH_3	$\nu_1^{DME}(a_1)$	3185.0	21.3	3186.8	19.3	1.8
	$\nu_2^{DME}(a_1)$	3028.3	65.4	3032.5	70.2	4.2
	$\nu_3^{DME}(a_1)$	1541.7	1.7	1541.3	1.2	-0.4
	$\nu_4^{DME}(a_1)$	1507.7	0.4	1507.1	0.2	-0.6
	$\nu_5^{DME}(a_1)$	1283.3	6.1	1285.1	5.8	1.8
	$\nu_6^{DME}(a_1)$	968.6	40.5	966.6	48.1	-2.0
	$\nu_7^{DME}(a_1)$	422.8	1.8	423.9	2.4	1.1
	$\nu_8^{DME}(a_2)$	3091.9	0.0	3098.5	0.1	6.6
	$\nu_9^{DME}(a_2)$	1498.0	0.0	1499.3	0.0	1.3
	$\nu_{10}^{DME}(a_2)$	1177.3	0.0	1177.9	0.0	0.6
	$\nu_{11}^{DME}(b_1)$	198.6	0.0	200.5	0.0	1.9
	$\nu_{12}^{DME}(b_1)$	3184.4	26.6	3186.0	16.7	1.6
	$\nu_{13}^{DME}(b_1)$	3020.3	53.9	3025.0	49.1	4.7
	$\nu_{14}^{DME}(b_1)$	1521.8	13.0	1521.4	15.4	-0.4
	$\nu_{15}^{DME}(b_1)$	1482.1	3.4	1481.2	0.3	-0.9
	$\nu_{16}^{DME}(b_1)$	1225.3	109.2	1222.1	96.7	-3.2
	$\nu_{17}^{DME}(b_1)$	1138.9	27.7	1137.9	27.3	-1.0
	$\nu_{18}^{DME}(b_2)$	3086.0	119.3	3092.7	108.5	6.7
	$\nu_{19}^{DME}(b_2)$	1508.4	13.7	1509.9	13.2	1.5
	$\nu_{20}^{DME}(b_2)$	1212.4	8.1	1213.4	8.6	1.0
	$\nu_{21}^{DME}(b_2)$	261.9	6.8	262.7	6.2	0.8

N_2O	$\nu_1^{N_2O}\left(\Sigma^+\right)$	1285.6	10.8	1292.6	10.9	7.0
	$\nu_{2a}^{N_2O}\left(\Pi\right)$	537.9	2.7	544.3	2.7	6.4
	$\nu_{2b}^{N_2O}\left(\Pi\right)$	537.9	2.7	536.3	5.7	-1.6
	$\nu_3^{N_2O}\left(\Sigma^-\right)$	2243.0	366.0	2255.8	321.4	12.8

Van der Waals modes				106.8	0.1	
				83.0	0.0	
				45.8	6.4	
				52.6	0.7	
				37.2	0.0	

Table S11. MP2/6-311++G(d,p) calculated harmonic vibrational frequencies, in cm^{-1}, and infrared intensities, , in km mol^{-1}, obtained for the tetrel bonded 1:1 complex of dimethyl ether with OCS. For completeness, also the shifts induced by the complexation with the partner molecule are given. Where applicable, BSSE corrected derivatives were used.

		Monomer		Complex		
	Assignment ·	$\tilde{\nu}$	Int ·	$\tilde{\nu}$	Int ·	$\Delta\tilde{\nu}$
CH_3OCH_3	$\nu_1^{DME}(a_1)$	3185.0	21.3	3187.6	16.9	2.6
	$\nu_2^{DME}(a_1)$	3028.3	65.4	3030.9	76.8	2.6
	$\nu_3^{DME}(a_1)$	1541.7	1.7	1540.5	0.8	-1.2
	$\nu_4^{DME}(a_1)$	1507.7	0.4	1506.8	0.2	-0.9
	$\nu_5^{DME}(a_1)$	1283.3	6.1	1284.7	5.3	1.4
	$\nu_6^{DME}(a_1)$	968.6	40.5	966.6	52.9	-2.0
	$\nu_7^{DME}(a_1)$	422.8	1.8	423.9	2.7	1.1
	$\nu_8^{DME}(a_2)$	3091.9	0.0	3096.2	0.2	4.3
	$\nu_9^{DME}(a_2)$	1498.0	0.0	1498.6	0.1	0.6
	$\nu_{10}^{DME}(a_2)$	1177.3	0.0	1177.4	0.0	0.1
	$\nu_{11}^{DME}(b_1)$	198.6	0.0	200.3	0.0	1.7
	$\nu_{12}^{DME}(b_1)$	3184.4	26.6	3183.1	20.5	-1.3
	$\nu_{13}^{DME}(b_1)$	3020.3	53.9	3023.2	49.5	2.9
	$\nu_{14}^{DME}(b_1)$	1521.8	13.0	1521.0	16.0	-0.8
	$\nu_{15}^{DME}(b_1)$	1482.1	3.4	1480.8	0.1	-1.3
	$\nu_{16}^{DME}(b_1)$	1225.3	109.2	1222.7	90.1	-2.6
	$\nu_{17}^{DME}(b_1)$	1138.9	27.7	1138.3	24.8	-0.6
	$\nu_{18}^{DME}(b_2)$	3086.0	119.3	3090.4	108.9	4.4
	$\nu_{19}^{DME}(b_2)$	1508.4	13.7	1509.5	12.1	1.1
	$\nu_{20}^{DME}(b_2)$	1212.4	8.1	1212.9	8.6	0.5
	$\nu_{21}^{DME}(b_2)$	261.9	6.8	262.4	5.8	0.5

OCS	$\nu_1^{COS}(\Sigma^+)$	901.2	5.8	901.5	5.7	0.3
	$\nu_{2a}^{COS}(\Pi)$	495.0	0.9	498.0	0.9	3.0

$\nu_{2b}^{COS}(\Pi)$	495.0	0.9	488.3	4.8	-6.7
$\nu_{3}^{COS}(\Sigma^{-})$	2100.7	669.1	2104.7	628.9	4.0

Van der Waals modes			82.7	0.1	
			67.3	0.2	
			41.9	0.8	
			37.4	5.6	
			25.6	0.7	

Table S12. MP2/6-311++G(d,p) calculated harmonic vibrational frequencies, in cm^{-1}, and infrared intensities, in km mol^{-1}, obtained for the chalcogen bonded 1:1 complex of dimethyl ether with OCS. For completeness, also the shifts induced by the complexation with the partner molecule are given. Where applicable, BSSE corrected derivatives were used.

		Monomer		Complex		
	Assignment	ν^a	Int^b	ν^a	Int^b	$\Delta\nu^a$
CH_3OCH_3	$\nu_1^{DME}(a_1)$	3185.0	21.3	3184.5	24.0	-0.5
	$\nu_2^{DME}(a_1)$	3028.3	65.4	3031.9	80.0	3.6
	$\nu_3^{DME}(a_1)$	1541.7	1.7	1541.2	0.4	-0.5
	$\nu_4^{DME}(a_1)$	1507.7	0.4	1508.0	13.1	0.3
	$\nu_5^{DME}(a_1)$	1283.3	6.1	1284.4	6.1	1.1
	$\nu_6^{DME}(a_1)$	968.6	40.5	965.3	64.0	-3.3
	$\nu_7^{DME}(a_1)$	422.8	1.8	422.8	3.1	0.0
	$\nu_8^{DME}(a_2)$	3091.9	0.0	3098.1	0.0	6.2
	$\nu_9^{DME}(a_2)$	1498.0	0.0	1497.5	0.0	-0.5
	$\nu_{10}^{DME}(a_2)$	1177.3	0.0	1176.9	0.0	-0.4
	$\nu_{11}^{DME}(b_1)$	198.6	0.0	196.9	0.0	-1.7
	$\nu_{12}^{DME}(b_1)$	3184.4	26.6	3183.8	22.1	-0.6
	$\nu_{13}^{DME}(b_1)$	3020.3	53.9	3024.7	51.4	4.4
	$\nu_{14}^{DME}(b_1)$	1521.8	13.0	1521.6	13.4	-0.2
	$\nu_{15}^{DME}(b_1)$	1482.1	3.4	1482.2	1.9	0.1
	$\nu_{16}^{DME}(b_1)$	1225.3	109.2	1222.8	103.2	-2.5
	$\nu_{17}^{DME}(b_1)$	1138.9	27.7	1138.3	29.0	-0.6
	$\nu_{18}^{DME}(b_2)$	3086.0	119.3	3092.3	106.5	6.3
	$\nu_{19}^{DME}(b_2)$	1508.4	13.7	1508.2	0.6	-0.2
	$\nu_{20}^{DME}(b_2)$	1212.4	8.1	1212.4	8.4	0.0
	$\nu_{21}^{DME}(b_2)$	261.9	6.8	260.3	6.1	-1.6
OCS	$\nu_1^{SCO}(\Sigma^+)$	901.2	5.8	894.7	1.5	-6.5

$\nu_{2a}^{SCO}\left(\Pi\right)$	495.0	0.9	504.2	0.9	9.2
$\nu_{2b}^{SCO}\left(\Pi\right)$	495.0	0.9	502.4	0.8	7.4
$\nu_{3}^{SCO}\left(\Sigma^{-}\right)$	2100.7	669.1	2089.3	737.8	-11.4
Van der Waals modes			55.9	0.4	
			51.0	0.8	
			41.0	3.5	
			20.1	3.1	
			5.1	0.4	

Table S13. MP2/6-311++G(d,p) calculated harmonic vibrational frequencies, in cm^{-1}, and infrared intensities, in km mol^{-1}, obtained for 1:1 complex of trimethyl amine with $^{12}CO_2$. For completeness, also the shifts induced by the complexation with the partner molecule are given. Where applicable, BSSE corrected derivatives were used.

		Monomer		Complex		
	Assignment	$\tilde{\nu}$	Int	$\tilde{\nu}$	Int	$\Delta\tilde{\nu}$
$N(CH_3)_3$	$\nu_1^{TMA}(a_1)$	3114.1	42.3	3116.2	39.1	2.1
	$\nu_2^{TMA}(a_1)$	2970.4	179.7	2980.3	178.4	9.9
	$\nu_3^{TMA}(a_1)$	1521.8	24.9	1522.6	26.9	0.8
	$\nu_4^{TMA}(a_1)$	1492.3	0.3	1492.8	0.2	0.5
	$\nu_5^{TMA}(a_1)$	1228.3	25.4	1232.3	24.3	4.0
	$\nu_6^{TMA}(a_1)$	860.8	25.8	859.5	27.8	-1.3
	$\nu_7^{TMA}(a_1)$	389.4	8.6	396.8	12.8	7.4
	$\nu_8^{TMA}(a_2)$	3164.9	0.0	3163.2	9.1	-1.7
	$\nu_9^{TMA}(a_2)$	1507.3	0.0	1506.9	0.0	-0.4
	$\nu_{10}^{TMA}(a_2)$	1076.3	0.0	1076.5	0.0	0.2
	$\nu_{11}^{TMA}(a_2)$	240.9	0.0	237.0	0.0	-3.9
	$\nu_{12a}^{TMA}(e)$	3168.0	35.4	3169.2	30.9	1.2
	$\nu_{12b}^{TMA}(e)$	3168.0	35.4	3167.6	18.8	-0.4
	$\nu_{13a}^{TMA}(e)$	3112.7	25.6	3115.2	16.3	2.5
	$\nu_{13b}^{TMA}(e)$	3112.7	25.6	3110.5	26.8	-2.2
	$\nu_{14a}^{TMA}(e)$	2962.1	46.1	2973.0	43.3	10.9
	$\nu_{14b}^{TMA}(e)$	2962.1	46.1	2973.0	42.7	10.9
	$\nu_{15a}^{TMA}(e)$	1529.5	9.7	1530.5	14.6	1.0
	$\nu_{15b}^{TMA}(e)$	1529.5	9.7	1529.1	10.8	-0.4
	$\nu_{16a}^{TMA}(e)$	1499.3	7.0	1500.3	5.2	1.0
	$\nu_{16b}^{TMA}(e)$	1499.3	7.0	1499.0	3.5	-0.3
	$\nu_{17a}^{TMA}(e)$	1454.5	0.5	1454.8	0.0	0.3
	$\nu_{17b}^{TMA}(e)$	1454.5	0.5	1453.9	0.1	-0.6
	$\nu_{18a}^{TMA}(e)$	1325.7	15.4	1325.4	12.5	-0.3

	$\nu_{18b}^{TMA}(e)$	1325.7	15.4	1323.1	14.3	-2.6
	$\nu_{19a}^{TMA}(e)$	1137.8	8.6	1136.9	6.9	-0.9
	$\nu_{19b}^{TMA}(e)$	1137.8	8.6	1136.6	8.1	-1.2
	$\nu_{20a}^{TMA}(e)$	1082.6	15.6	1081.0	15.7	-1.6
	$\nu_{20b}^{TMA}(e)$	1082.6	15.6	1081.0	14.4	-1.6
	$\nu_{21a}^{TMA}(e)$	423.4	0.0	423.0	0.0	-0.4
	$\nu_{21b}^{TMA}(e)$	423.4	0.0	422.8	0.0	-0.6
	$\nu_{22a}^{TMA}(e)$	289.4	0.5	290.2	0.6	0.8
	$\nu_{22b}^{TMA}(e)$	289.4	0.5	284.6	0.4	-4.8
$^{12}CO_2$	$\nu_1^{^{12}CO_2}(\Sigma_g^+)$	1335.4	0.0	1334.8	1.0	-0.6
	$\nu_{2a}^{^{12}CO_2}(\Pi_g)$	655.2	24.7	661.1	19.0	5.9
	$\nu_{2b}^{^{12}CO_2}(\Pi_u)$	655.2	24.7	625.0	67.9	-30.2
	$\nu_3^{^{12}CO_2}(\Sigma_u^+)$	2432.7	603.9	2429.2	500.0	-3.5
Van der Waals modes				127.5	0.2	
				75.0	0.4	
				42.6	0.1	
				40.5	0.1	
				7.0	0.0	

Table S14. MP2/6-311++G(d,p) calculated harmonic vibrational frequencies, in cm^{-1}, and infrared intensities, in km mol^{-1}, obtained for the 1:1 complex of trimethyl ether with $^{13}CO_2$. For completeness, also the shifts induced by the complexation with the partner molecule are given. Where applicable, BSSE corrected derivatives were used.

		Monomer		Complex		
	Assignment	$\tilde{\nu}$	Int	$\tilde{\nu}$	Int	$\Delta\tilde{\nu}$
$N(CH_3)_3$	ν_1^{TMA} (a_1)	3114.1	42.3	3116.2	39.1	2.1
	ν_2^{TMA} (a_1)	2970.4	179.7	2980.3	178.4	9.9
	ν_3^{TMA} (a_1)	1521.8	24.9	1522.6	26.8	0.8
	ν_4^{TMA} (a_1)	1492.3	0.3	1492.8	0.2	0.5
	ν_5^{TMA} (a_1)	1228.3	25.4	1232.3	24.3	4.0
	ν_6^{TMA} (a_1)	860.8	25.8	859.4	28.1	-1.4
	ν_7^{TMA} (a_1)	389.4	8.6	396.8	12.9	7.4
	ν_8^{TMA} (a_2)	3164.9	0.0	3163.2	9.0	-1.7
	ν_9^{TMA} (a_2)	1507.3	0.0	1506.9	0.0	-0.4
	ν_{10}^{TMA} (a_2)	1076.3	0.0	1076.5	0.0	0.2
	ν_{11}^{TMA} (a_2)	240.9	0.0	237.0	0.0	-3.9
	ν_{12a}^{TMA} (e)	3168.0	35.4	3169.2	30.9	1.2
	ν_{12b}^{TMA} (e)	3168.0	35.4	3167.6	18.7	-0.4
	ν_{13a}^{TMA} (e)	3112.7	25.6	3115.2	16.2	2.5
	ν_{13b}^{TMA} (e)	3112.7	25.6	3110.5	26.8	-2.2
	ν_{14a}^{TMA} (e)	2962.1	46.1	2973.0	43.3	10.9
	ν_{14b}^{TMA} (e)	2962.1	46.1	2973.0	42.7	10.9
	ν_{15a}^{TMA} (e)	1529.5	9.7	1530.4	14.7	0.9
	ν_{15b}^{TMA} (e)	1529.5	9.7	1529.1	10.8	-0.4
	ν_{16a}^{TMA} (e)	1499.3	7.0	1500.3	5.2	1.0
	ν_{16b}^{TMA} (e)	1499.3	7.0	1499.0	3.4	-0.3
	ν_{17a}^{TMA} (e)	1454.5	0.5	1454.8	0.0	0.3
	ν_{17b}^{TMA} (e)	1454.5	0.5	1453.9	0.1	-0.6
	ν_{18a}^{TMA} (e)	1325.7	15.4	1325.4	12.5	-0.3

	$\nu_{18b}^{TMA}(e)$	1325.7	15.4	1323.1	14.3	-2.6
	$\nu_{19a}^{TMA}(e)$	1137.8	8.6	1136.9	6.9	-0.9
	$\nu_{19b}^{TMA}(e)$	1137.8	8.6	1136.6	8.1	-1.2
	$\nu_{20a}^{TMA}(e)$	1082.6	15.6	1081.0	15.7	-1.6
	$\nu_{20b}^{TMA}(e)$	1082.6	15.6	1081.0	14.4	-1.6
	$\nu_{21a}^{TMA}(e)$	423.4	0.0	423.0	0.0	-0.4
	$\nu_{21b}^{TMA}(e)$	423.4	0.0	422.8	0.0	-0.6
	$\nu_{22a}^{TMA}(e)$	289.4	0.5	290.2	0.6	0.8
	$\nu_{22b}^{TMA}(e)$	289.4	0.5	284.6	0.4	-4.8
$^{13}CO_2$	$\nu_1^{^{13}CO_2}(\Sigma_g^+)$	1335.4	0.0	1334.8	0.9	-0.6
	$\nu_{2a}^{^{13}CO_2}(\Pi_g)$	636.5	23.3	642.1	18.0	5.6
	$\nu_{2b}^{^{13}CO_2}(\Pi_u)$	636.5	23.3	607.7	63.5	-28.8
	$\nu_3^{^{13}CO_2}(\Sigma_u^+)$	2363.5	570.0	2359.9	472.6	-3.6
Van der Waals modes				127.5	0.2	
				75.0	0.4	
				42.6	0.1	
				40.5	0.1	
				7.0	0.0	

Table S15. MP2/6-311++G(d,p) calculated harmonic vibrational frequencies, in cm^{-1}, and infrared intensities, in km mol^{-1}, obtained for the 1:1 complex of trimethyl amine with N_2O. For completeness, also the shifts induced by the complexation with the partner molecule are given. Where applicable, BSSE corrected derivatives were used.

	Assignment	Monomer $\tilde{\nu}$	Monomer Int	Complex $\tilde{\nu}$	Complex Int	$\Delta\tilde{\nu}$
$N(CH_3)_3$	$\nu_1^{TMA}(a_1)$	3114.1	42.3	3108.6	28.5	-5.5
	$\nu_2^{TMA}(a_1)$	2970.4	179.7	2976.4	179.6	6.0
	$\nu_3^{TMA}(a_1)$	1521.8	24.9	1522.3	27.3	0.5
	$\nu_4^{TMA}(a_1)$	1492.3	0.3	1492.6	0.4	0.3
	$\nu_5^{TMA}(a_1)$	1228.3	25.4	1230.6	24.7	2.3
	$\nu_6^{TMA}(a_1)$	860.8	25.8	859.6	28.6	-1.2
	$\nu_7^{TMA}(a_1)$	389.4	8.6	393.4	9.2	4.0
	$\nu_8^{TMA}(a_2)$	3164.9	0.0	3161.5	9.6	-3.4
	$\nu_9^{TMA}(a_2)$	1507.3	0.0	1507.0	0.0	-0.3
	$\nu_{10}^{TMA}(a_2)$	1076.3	0.0	1076.3	0.0	0.0
	$\nu_{11}^{TMA}(a_2)$	240.9	0.0	239.1	0.0	-1.8
	$\nu_{12a}^{TMA}(e)$	3168.0	35.4	3167.8	32.5	-0.2
	$\nu_{12b}^{TMA}(e)$	3168.0	35.4	3166.2	17.6	-1.8
	$\nu_{13a}^{TMA}(e)$	3112.7	25.6	3114.4	38.2	1.7
	$\nu_{13b}^{TMA}(e)$	3112.7	25.6	3113.3	18.8	0.6
	$\nu_{14a}^{TMA}(e)$	2962.1	46.1	2969.0	45.0	6.9
	$\nu_{14b}^{TMA}(e)$	2962.1	46.1	2968.4	44.3	6.3
	$\nu_{15a}^{TMA}(e)$	1529.5	9.7	1530.1	15.4	0.6
	$\nu_{15b}^{TMA}(e)$	1529.5	9.7	1529.2	10.3	-0.3
	$\nu_{16a}^{TMA}(e)$	1499.3	7.0	1500.2	5.1	0.9
	$\nu_{16b}^{TMA}(e)$	1499.3	7.0	1498.9	3.7	-0.4
	$\nu_{17a}^{TMA}(e)$	1454.5	0.5	1454.4	0.0	-0.1
	$\nu_{17b}^{TMA}(e)$	1454.5	0.5	1453.6	0.2	-1.1

	$\nu_{18a}^{TMA}(e)$	1325.7	15.4	1325.3	12.9	-0.4
	$\nu_{18b}^{TMA}(e)$	1325.7	15.4	1323.9	14.8	-1.8
	$\nu_{19a}^{TMA}(e)$	1137.8	8.6	1137.2	7.0	-0.6
	$\nu_{19b}^{TMA}(e)$	1137.8	8.6	1136.7	8.3	-1.1
	$\nu_{20a}^{TMA}(e)$	1082.6	15.6	1081.9	15.0	-0.7
	$\nu_{20b}^{TMA}(e)$	1082.6	15.6	1081.3	15.0	-1.3
	$\nu_{21a}^{TMA}(e)$	423.4	0.0	423.2	0.0	-0.2
	$\nu_{21b}^{TMA}(e)$	423.4	0.0	422.7	0.0	-0.7
	$\nu_{22a}^{TMA}(e)$	289.4	0.5	290.1	0.6	0.7
	$\nu_{22b}^{TMA}(e)$	289.4	0.5	286.6	0.4	-2.8
N_2O	$\nu_{1}^{N_2O}(\Sigma^+)$	1285.6	10.8	1291.3	8.9	5.7
	$\nu_{2a}^{N_2O}(\Pi)$	537.9	2.7	543.3	2.3	5.4
	$\nu_{2b}^{N_2O}(\Pi)$	537.9	2.7	525.3	6.5	-12.6
	$\nu_{3}^{N_2O}(\Sigma^-)$	2243.0	366.0	2254.1	296.0	11.1
Van der Waals modes				102.8	0.3	
				72.3	0.0	
				36.9	0.2	
				31.9	0.1	
				7.3	0.1	

Table S16. MP2/6-311++G(d,p) calculated harmonic vibrational frequencies, in cm^{-1}, and infrared intensities, in km mol^{-1}, obtained for the tetrel bonded 1:1 complex of dimethyl ether with OCS. For completeness, also the shifts induced by the complexation with the partner molecule are given. Where applicable, BSSE corrected derivatives were used.

		Monomer		Complex		
	Assignment ·	$\tilde{\nu}$	Int ·	$\tilde{\nu}$	Int ·	$\Delta\tilde{\nu}$
$N(CH_3)_3$	$\nu_1^{TMA}(a_1)$	3114.1	42.3	3109.9	22.1	-4.2
	$\nu_2^{TMA}(a_1)$	2970.4	179.7	2973.9	200.0	3.5
	$\nu_3^{TMA}(a_1)$	1521.8	24.9	1521.8	27.7	0.0
	$\nu_4^{TMA}(a_1)$	1492.3	0.3	1492.3	0.4	0.0
	$\nu_5^{TMA}(a_1)$	1228.3	25.4	1229.5	24.1	1.2
	$\nu_6^{TMA}(a_1)$	860.8	25.8	859.5	33.6	-1.3
	$\nu_7^{TMA}(a_1)$	389.4	8.6	390.5	11.8	1.1
	$\nu_8^{TMA}(a_2)$	3164.9	0.0	3162.4	1.5	-2.5
	$\nu_9^{TMA}(a_2)$	1507.3	0.0	1506.8	0.0	-0.5
	$\nu_{10}^{TMA}(a_2)$	1076.3	0.0	1076.5	0.0	0.2
	$\nu_{11}^{TMA}(a_2)$	240.9	0.0	239.2	0.0	-1.7
	$\nu_{12a}^{TMA}(e)$	3168.0	35.4	3166.5	27.5	-1.5
	$\nu_{12b}^{TMA}(e)$	3168.0	35.4	3165.1	28.7	-2.9
	$\nu_{13a}^{TMA}(e)$	3112.7	25.6	3112.6	39.1	-0.1
	$\nu_{13b}^{TMA}(e)$	3112.7	25.6	3111.0	23.4	-1.7
	$\nu_{14a}^{TMA}(e)$	2962.1	46.1	2966.4	45.5	4.3
	$\nu_{14b}^{TMA}(e)$	2962.1	46.1	2965.8	44.9	3.7
	$\nu_{15a}^{TMA}(e)$	1529.5	9.7	1529.6	17.3	0.1
	$\nu_{15b}^{TMA}(e)$	1529.5	9.7	1529.3	11.4	-0.2
	$\nu_{16a}^{TMA}(e)$	1499.3	7.0	1499.7	4.6	-0.4
	$\nu_{16b}^{TMA}(e)$	1499.3	7.0	1498.3	2.3	-1.0
	$\nu_{17a}^{TMA}(e)$	1454.5	0.5	1453.9	0.1	-0.6
	$\nu_{17b}^{TMA}(e)$	1454.5	0.5	1453.6	0.2	-0.9
	$\nu_{18a}^{TMA}(e)$	1325.7	15.4	1326.7	12.1	1.0

	$\nu_{18b}^{TMA}(e)$	1325.7	15.4	1325.3	14.6	-0.4
	$\nu_{19a}^{TMA}(e)$	1137.8	8.6	1136.8	8.1	-1.0
	$\nu_{19b}^{TMA}(e)$	1137.8	8.6	1136.4	7.2	-1.4
	$\nu_{20a}^{TMA}(e)$	1082.6	15.6	1083.0	13.4	0.4
	$\nu_{20b}^{TMA}(e)$	1082.6	15.6	1082.4	15.1	-0.2
	$\nu_{21a}^{TMA}(e)$	423.4	0.0	423.4	0.1	0.0
	$\nu_{21b}^{TMA}(e)$	423.4	0.0	422.9	0.0	-0.5
	$\nu_{22a}^{TMA}(e)$	289.4	0.5	288.6	0.6	-0.8
	$\nu_{22b}^{TMA}(e)$	289.4	0.5	286.3	0.5	-3.1
OCS	$\nu_{1}^{COS}(\Sigma^{+})$	901.2	5.8	900.3	5.5	-0.9
	$\nu_{2a}^{COS}(\Pi)$	495.0	0.9	499.5	0.6	4.5
	$\nu_{2b}^{COS}(\Pi)$	495.0	0.9	478.0	7.9	-17.0
	$\nu_{3}^{COS}(\Sigma^{-})$	2100.7	669.1	2101.2	595.8	0.5
Van der Waals modes				86.1	0.3	
				63.6	0.0	
				38.2	0.2	
				35.5	0.2	
				5.1	0.3	

Table S17. MP2/6-311++G(d,p) calculated harmonic vibrational frequencies, in cm^{-1}, and infrared intensities, in km mol^{-1}, obtained for the chalcogen bonded 1:1 complex of dimethyl ether with OCS. For completeness, also the shifts induced by the complexation with the partner molecule are given. Where applicable, BSSE corrected derivatives were used.

		Monomer		Complex		
	Assignment ·	$\tilde{\nu}$	Int ·	$\tilde{\nu}$	Int ·	$\Delta\tilde{\nu}$
$N(CH_3)_3$	$\nu_1^{TMA}(a_1)$	3114.1	42.3	3113.5	46.3	-0.6
	$\nu_2^{TMA}(a_1)$	2970.4	179.7	2976.4	208.5	6.0
	$\nu_3^{TMA}(a_1)$	1521.8	24.9	1521.2	31.0	-0.6
	$\nu_4^{TMA}(a_1)$	1492.3	0.3	1492.7	0.1	0.4
	$\nu_5^{TMA}(a_1)$	1228.3	25.4	1230.0	24.4	1.7
	$\nu_6^{TMA}(a_1)$	860.8	25.8	858.3	39.8	-2.5
	$\nu_7^{TMA}(a_1)$	389.4	8.6	392.0	14.3	2.6
	$\nu_8^{TMA}(a_2)$	3164.9	0.0	3163.3	0.5	-1.6
	$\nu_9^{TMA}(a_2)$	1507.3	0.0	1506.9	0.0	-0.4
	$\nu_{10}^{TMA}(a_2)$	1076.3	0.0	1076.0	0.0	-0.3
	$\nu_{11}^{TMA}(a_2)$	240.9	0.0	236.2	0.0	-4.7
	$\nu_{12a}^{TMA}(e)$	3168.0	35.4	3166.9	31.8	-1.1
	$\nu_{12b}^{TMA}(e)$	3168.0	35.4	3166.2	31.3	-1.8
	$\nu_{13a}^{TMA}(e)$	3112.7	25.6	3112.2	24.6	-0.5
	$\nu_{13b}^{TMA}(e)$	3112.7	25.6	3111.7	20.2	-1.0
	$\nu_{14a}^{TMA}(e)$	2962.1	46.1	2969.1	43.8	7.0
	$\nu_{14b}^{TMA}(e)$	2962.1	46.1	2968.8	44.7	6.7
	$\nu_{15a}^{TMA}(e)$	1529.5	9.7	1529.6	11.2	0.1
	$\nu_{15b}^{TMA}(e)$	1529.5	9.7	1529.6	11.7	0.1
	$\nu_{16a}^{TMA}(e)$	1499.3	7.0	1498.6	5.6	-0.7
$N(CH_3)_3$	$\nu_{16b}^{TMA}(e)$	1499.3	7.0	1498.4	4.9	-0.9
	$\nu_{17a}^{TMA}(e)$	1454.5	0.5	1454.3	0.2	-0.2
	$\nu_{17b}^{TMA}(e)$	1454.5	0.5	1454.2	0.1	-0.3

	$\nu_{18a}^{TMA}(e)$	1325.7	15.4	1325.2	14.6	-0.5
	$\nu_{18b}^{TMA}(e)$	1325.7	15.4	1324.8	14.7	-0.9
	$\nu_{19a}^{TMA}(e)$	1137.8	8.6	1136.3	8.0	-1.5
	$\nu_{19b}^{TMA}(e)$	1137.8	8.6	1136.0	7.9	-1.8
	$\nu_{20a}^{TMA}(e)$	1082.6	15.6	1081.7	15.5	-0.9
	$\nu_{20b}^{TMA}(e)$	1082.6	15.6	1081.5	15.4	-1.1
	$\nu_{21a}^{TMA}(e)$	423.4	0.0	422.6	0.0	-0.8
	$\nu_{21b}^{TMA}(e)$	423.4	0.0	422.5	0.0	-0.9
	$\nu_{22a}^{TMA}(e)$	289.4	0.5	285.3	0.5	-4.1
	$\nu_{22b}^{TMA}(e)$	289.4	0.5	285.2	0.5	-4.2
OCS	$\nu_{1}^{SCO}(\Sigma^{+})$	901.2	5.8	888.0	2.5	-13.2
	$\nu_{2a}^{SCO}(\Pi)$	495.0	0.9	506.2	1.1	11.2
	$\nu_{2b}^{SCO}(\Pi)$	495.0	0.9	506.0	1.1	11.0
	$\nu_{3}^{SCO}(\Sigma^{-})$	2100.7	669.1	2085.2	755.4	-15.5
Van der Waals modes				57.0	0.3	
				53.1	0.3	
				52.9	0.3	
				11.6	0.0	
				7.4	0.0	
